# Alpine peak pressure and tectono-metamorphic history of the Monte Rosa nappe: evidence from the cirque du Véraz, upper Ayas valley, Italy

**DOI:** 10.1186/s00015-021-00397-3

**Published:** 2021-10-29

**Authors:** Joshua D. Vaughan-Hammon, Cindy Luisier, Lukas P. Baumgartner, Stefan M. Schmalholz

**Affiliations:** 1 Institute of Earth Sciences, University of Lausanne, 1015 Lausanne, Switzerland; 2 CNRS, Géosciences Rennes UMR 6118, University of Rennes, Rennes, France

**Keywords:** Monte Rosa nappe, Alpine peak pressure, Whiteschist, Tectonic pressure, Mg-chloritoid

## Abstract

The Monte Rosa nappe consists of a wide range of lithologies that record conditions associated with peak Alpine metamorphism. While peak temperature conditions inferred from previous studies largely agree, variable peak pressures have been estimated for the Alpine high-pressure metamorphic event. Small volumes of whiteschist lithologies with the assemblage chloritoid + phengite + talc + quartz record peak pressures up to 0.6 GPa higher compared to associated metapelitic and metagranitic lithologies, which yield a peak pressure of ca. 1.6 GPa. The reason for this pressure difference is disputed, and proposed explanations include tectonic mixing of rocks from different burial depths (mélange) or local deviations of the pressure from the lithostatic value caused by heterogeneous stress conditions between rocks of contrasting mechanical properties.

We present results of detailed field mapping, structural analysis and a new geological map for a part of the Monte Rosa nappe exposed at the cirque du Véraz field area (head of the Ayas valley, Italy). Results of the geological mapping and structural analysis shows the structural coherency within the western portions of the Monte Rosa nappe. This structural coherency falsifies the hypothesis of a tectonic mélange as reason for peak pressure variations. Structural analysis indicates two major Alpine deformation events, in agreement with earlier studies: (1) north-directed nappe emplacement, and (2) south-directed backfolding.

We also analyze a newly discovered whiteschist body, which is located at the intrusive contact between Monte Rosa metagranite and surrounding metapelites. This location is different to previous whiteschist occurrences, which were entirely embedded within metagranite. Thermodynamic calculations using metamorphic assemblage diagrams resulted in 2.1 ± 0.2 GPa and 560 ± 20 °C for peak Alpine metamorphic conditions. These results agree with metamorphic conditions inferred for previously investigated nearby whiteschist outcrops embedded in metagranite. The new results, hence, confirm the peak pressure differences between whiteschists and the metagranite and metapelite. To better constrain the prograde pressure–temperature history of the whiteschist, we compare measured Mg zoning in chloritoid with Mg zoning predicted by fractional crystallization pseudo-section modelling for several hypothetical pressure–temperature paths. In order to reach a ca. 0.6 GPa higher peak pressure compared to the metapelite and metagranite, our results suggest that the whiteschist likely deviated from the prograde burial path recorded in metapelite and metagranite lithologies. However, the exact conditions at which the whiteschist pressure deviated are still contentious due to the strong temperature dependency of Mg partitioning in whiteschist assemblages. Our pseudo-section results suggest at least that there was no dramatic isothermal pressure increase recorded in the whiteschist.

## Introduction

**Fig. 1:**
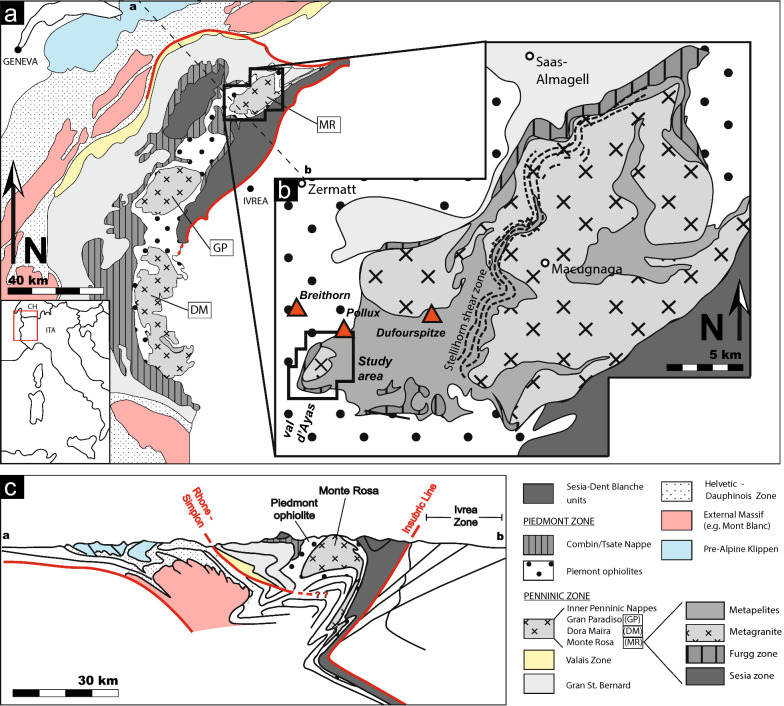
**a** Simplified geological tectonic map of the Western Alpine chain; adapted from Beltrando et al. (2010). **b** Simplified geological map of the Monte Rosa nappe; modified after Steck et al. (2015). **c** Simplified tectonic cross-section of the Western Alpine chain; modified after Steck et al. (2015)

**Fig. 2:**
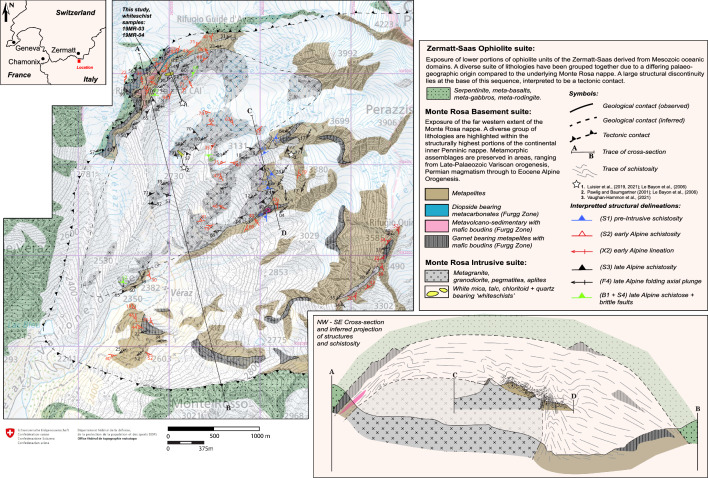
Geological map of exposure and cross-section of the Monte Rosa nappe and Zermatt-Saas nappe at the upper val d’Ayas, Italy. Location of previous studies on peak Alpine metamorphism in the area are indicated (white stars)

The complex history of Alpine tectonic activity can be observed in the Monte Rosa region of the Central Alps (Fig. 1). Here, the former Piedmont oceanic domain separates the continental units of the former Europe-derived southern Briançonnais margin (Monte Rosa nappe) from the Sesia-Dent Blanche units derived from the Adriatic margin (Fig. 1) (Dal Piaz, 2001; Dal Piaz et al., 2001; De Graciansky et al., 2011; Steck et al., 2015). A spectacular region to view the present-day juxtaposition of these geological domains is found at the grand cirque du Véraz at the head of the Ayas valley, Aosta region, Italy (Figs. 1b and 2). The area exposes portions of the Penninic nappe stack involving the crustal rocks of the former Briançonnais domain: the Monte Rosa nappe, consisting of Variscan and older paragneisses intruded by Permian-aged granites (269 ± 4 Ma; (Pawlig, 2001). Structurally above the Monte Rosa nappe, to the north, is the Piedmont domain (Zermatt Saas and Combin units), comprising remnants of an oceanic domain of predominantly serpentinites (Lapen et al., 2007; e.g. Angiboust et al., 2009; McCarthy et al., 2020), as well as eclogitized metagabbros and metabasalts with locally well-preserved pillow structures, making up most of the Breithorn plateau including the Pollux, Rocca Nera and Klein Matterhorn peaks (Fig. 1b). The former Piedmont oceanic domain was bounded by hyper-extended magma-poor passive margins (e.g. Decandia, 1972; Manatschal & Müntener, 2009), but the size and geodynamic characteristics of the former Piedmont oceanic domain are disputed. Interpretations range from a wider ocean (> ca. 500 km) with mature oceanic crust having typically a thickness of ca. 6 km (e.g. Stampfli et al., 1998) to a narrower oceanic domain (< ca. 300 km) with only “embryonic” oceanic crust having a thickness of only a few kilometers due to ultra-slow spreading (e.g. McCarthy et al., 2020). Finally, continuing northwards and structurally up the sequence, above the Zermatt-Saas unit the Combin unit and eventually the Adria-derived continental unit of the Dent-Blanche nappe are overthrust (Bucher et al., 2003a; Compagnoni, 1977; Dal Piaz, 1999; Manzotti et al., 2017; Steck et al., 2015), that comprise the spectacular Matterhorn peak.

Here, we present a detailed geological map and cross-section of the upper portions of the Monte Rosa nappe exposed at the grand cirque du Véraz at the head of the Ayas valley, Italy (Fig. 2). The overall scope of this study aims to outline the key lithologies and structural features found here, not only through our investigations but also from work undertaken in the last century in the area (Bearth, 1952, 1958; Chopin & Monié, 1984; Dal Piaz, 2001; Dal Piaz et al., 2004; Darbellay, 2005; Dessimoz, 2005; Giacomazzi, 2017; Kramer, 2002; Luisier et al., 2019, 2021; Marger et al., 2019; Pawlig, 2001; Pawlig & Baumgartner, 2001). The structural evolution of the Monte Rosa nappe exposed at the grand cirque du Véraz is also compared with other studies throughout the nappe (Keller & Schmid, 2001; Kramer, 2002; e.g. Keller et al., 2004; Steck et al., 2015). Furthermore, due to the variety of lithologies and structures found here, as well as the complex poly-metamorphic history recorded, encompassing both Variscan and Alpine orogenesis, we outline a comprehensive geological history for this region of the Monte Rosa nappe. Specific attention is given to the tectono-metamorphic history during Alpine orogenesis.

The Monte Rosa basement exposed at the cirque du Véraz documents variable metamorphic conditions, specifically during peak Alpine high-pressure metamorphism. These variable metamorphic conditions were highlighted by discussions on peak pressure (P) estimates (Luisier et al., 2019; Vaughan-Hammon et al., 2021), but also published peak temperature (T) estimates are highly variable. A wide range of calculated peak metamorphic conditions has prompted the discussion concerning variations in estimated peak pressures in the Monte Rosa nappe. Published P estimates range between 1.2 and 2.7 GPa and T estimates range between 490 and 640 °C (Borghi et al., 1996; Chopin & Monié, 1984; Dal Piaz & Lombardo, 1986; Ferrando et al., 2002; Gasco et al., 2011; Keller et al., 2004; Lapen et al., 2007; Le Bayon et al., 2006). The maximum metamorphic P conditions recorded for the Monte Rosa nappe primarily involve minor volumes of lithologies termed ‘whiteschists’ that contain talc, chloritoid, phengite and quartz, indicating 2.4 GPa (Le Bayon et al., 2006) and ca. 2.2 GPa (Luisier et al. (2019), as well as mafic boudins indicating ca. 2.7 GPa (e.g. Gasco et al., 2011). In the cirque du Véraz, recent work has highlighted large disparities in peak Alpine P between whiteschist (ca. 2.2 GPa) and Monte Rosa metagranite (1.4 to 1.6 GPa) (Luisier et al. (2019) as well as metapelitic (1.6 ± 0.2 GPa) assemblages (Vaughan-Hammon et al., 2021), resulting in a potential P difference, ∆P, of 0.6 ± 0.2 GPa for the same peak Alpine metamorphic event.

Five possible hypotheses have been discussed in the literature to explain such P differences: (a) assemblages recording lower P were unable to equilibrate at high P conditions due to sluggish kinetics (e.g. Spear & Pattison, 2017), (b) high P indicators were lost during retrogression (Luisier et al., 2019), (c) differences in thermodynamic databases, solid-solution model and model assumptions (e.g., Fe^3+^ content or H_2_O activity) between studies result in artificial ∆P (Luisier et al., 2019), (d) tectonic mixing of rocks from different maximal Alpine burial depth, i.e. tectonic mélange (proposed for the Internal Penninic units of the western Alps, e.g., Dora Maira; (Dobretsov, 1991), and (e) the existence of local P deviations from the lithostatic value caused by differential stresses (Luisier et al., 2019).

Recent studies by Luisier et al. (2019) and Vaughan-Hammon et al. (2021) suggest to exclude (a) sluggish kinetics (e.g., because of measurements of high H_2_O content in phengite and the presence of prograde hydrous minerals, such as phengite and zoisite in plagioclase pseudomorphs in the metagranite), (b) retrogression of high P minerals (e.g., because of observations of the preservation of the original lamellar twinning in igneous plagioclase by albite pseudomorphing igneous plagioclase in metagranites) and (c) differences in thermodynamic databases (e.g., because metagranite and metapelite provide the same, within error, peak P estimates of ca. 1.6 GPa using the same database) as reasons for the observed metamorphic P variations between whiteschist (ca. 2.2 GPa) and metapelite as well as metagranite (both ca. 1.6 GPa). These studies also suggest, based on local field observations, that (d) tectonic mélange is unlikely the reason for the P variations. One aim of our study is to thoroughly test hypothesis (d) with detailed geological mapping. The suggested exclusion of explanations (a), (b) and (c) highlights the possibility that the reported peak P variations could have been caused by (e) local P deviations from the lithostatic P. Another aim of this study is to provide more estimates of Alpine peak P by analyzing a newly discovered whiteschist and to test whether the reported peak P variations between whiteschist and metagranite/metapelite persist.

For clarity, we briefly provide here some definitions and fundamental characteristics of stress and P in the context of geodynamics. For a lithostatic state of stress, all three principal stresses are identical and are controlled only by the gravitational acceleration, density and thickness of the overlying rock (e.g. Turcotte & Schubert, 2002). The mean stress of these identical principal stresses is, hence, equal to these principal stresses and is commonly termed lithostatic P (e.g. Turcotte & Schubert, 2002). It is well established that the state of stress cannot be lithostatic everywhere in the crust, as otherwise (i) discrete continental plates, (ii) continental plateaus or (iii) mountains would not exist over geological time scales (e.g. Jeffreys, 1937; Molnar & Lyon-Caen, 1988; Schmalholz et al., 2014; Turcotte & Schubert, 2002). To maintain lateral topographic variations of the Earth surface, for example between continents and oceans or between mountains and neighboring lowlands, differential stresses (up to ca. 100 MPa in average vertically across the lithosphere, (Schmalholz et al., 2019) are required, which means that the three principal stresses are not identical (Molnar & Lyon-Caen, 1988; Schmalholz et al., 2014, 2019; Turcotte & Schubert, 2002). Differential stresses are also intrinsically linked to plate driving forces, e.g. ridge push and slab pull, and are, hence, essential to drive continental collision and mountain building. For a differential state of stress, the P, or mean stress, is typically not the lithostatic P since usually the vertical principal stress is close to the lithostatic P, but one horizontal principal stress is either significantly smaller (e.g. during extension) or larger (e.g. during compression) than the lithostatic P (e.g. Sibson, 1974). Hence, for a differential state of stress, pressure—interpreted to be the mean stress—is typically different to the lithostatic P. The P for a differential state of stress is sometimes termed dynamic P (e.g. Gerya, 2015). If this dynamic P is larger than the lithostatic P, then the P difference is commonly termed tectonic overpressure, and if this dynamic P is smaller than the lithostatic P, the P difference is termed tectonic underpressure (e.g. Gerya, 2015; Mancktelow, 2008). In summary, a lithostatic state of stress is physically impossible everywhere within the Earth’s crust, especially during collisional mountain building. The main questions concerning deviations from the lithostatic P are: how large are these P deviations locally in the crust, and are these P deviations recorded and observable in the metamorphic rock record?

Here, we aim to contribute to the ongoing discussion of peak Alpine P variations recorded between whiteschist, Monte Rosa metagranite and metapelitic lithologies, by analyzing a newly discovered outcrop of whiteschist. Detailed petrological investigations of two samples of whiteschist from this new outcrop have been undertaken, enabling a further constraint on the peak Alpine metamorphic conditions incurred by the Monte Rosa nappe. We compare observed zoning of Mg in chloritoid whiteschists with Mg zoning predicted by fractional crystallization pseudo-section modelling for a range of prograde pathways to further constrain the P–T history during peak Alpine metamorphism. We test whether the newly discovered whiteschist confirms, or not, the estimated peak P of ca. 2.2 GPa for a different whiteschist outcrop (Luisier et al., 2019), which is located ca. 100 m away from the newly discovered outcrop.

## Petrological overview

### Geological setting

The Monte Rosa nappe represents one amongst the three major internal crystalline massifs of the Western and Central Alpine domain, along with the Dora Maira and Gran Paradiso massifs (Fig. 1a). The Monte Rosa nappe consists of a pre-Variscan suite, which became intruded by Permian-age granitic to granodiorite bodies [269 ± 4 Ma (Pawlig, 2001)], which we refer to as Monte Rosa granitoids, and was subsequently incorporated into the Alpine orogeny (Fig. 1b). A geological map is presented in Fig. 2 showing the position and separations of the main lithological units within the western portions of the Monte Rosa nappe exposed at the grand cirque du Véraz (upper Ayas valley, Italy). A detailed map of the upper Ayas valley has also been presented in Dal Piaz et al. (2004) with focus on Zermatt-Saas ophiolites in the western portions of the valley. The following section outlines the main field relations and petrological characteristics of lithologies belonging to the Monte Rosa nappe. The structural features of these lithologies are summarized in Sect. 3.

### Lithological descriptions

#### Polymetamorphic basement complex

**Fig. 3:**
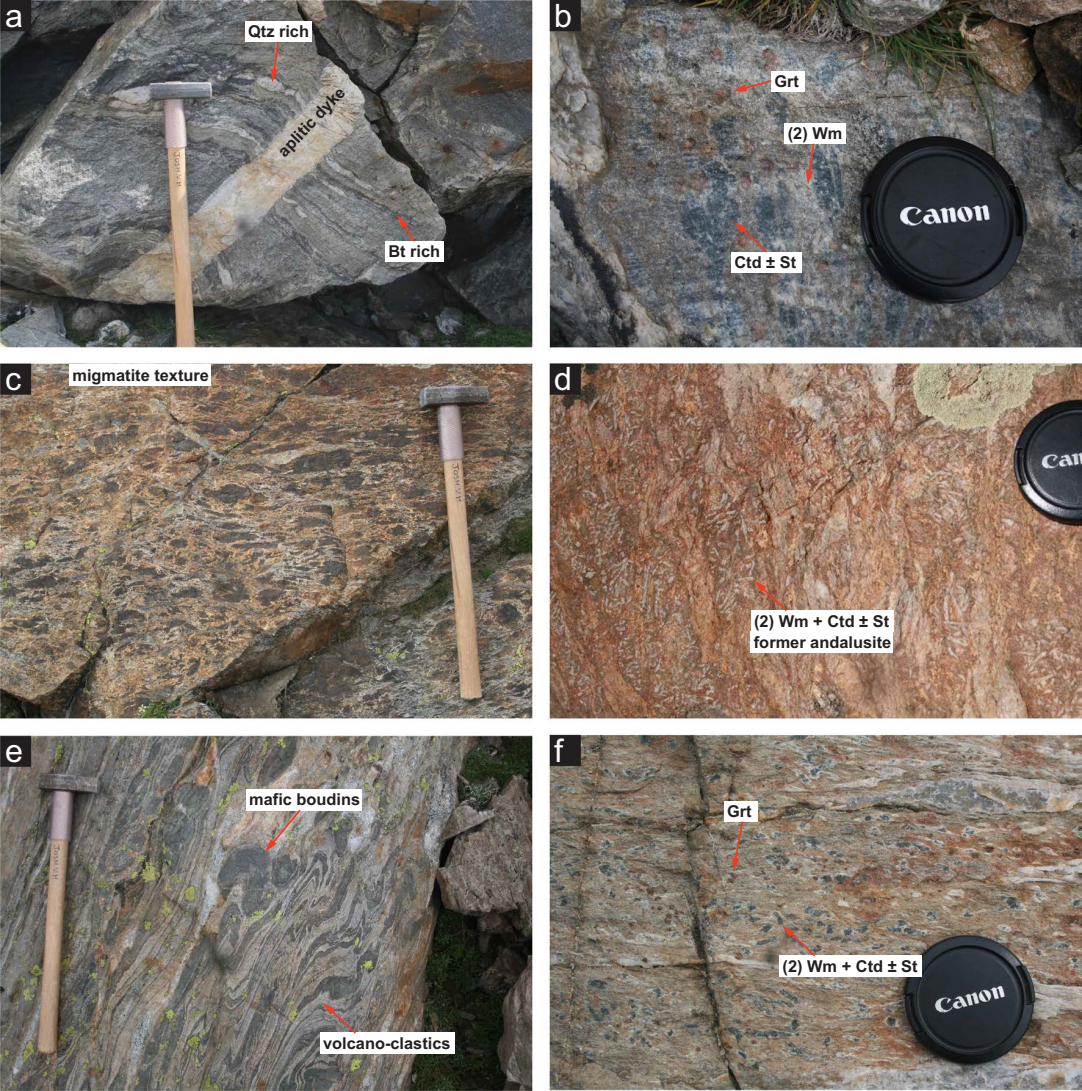
Mesoscopic aspect of some key lithologies belonging to the Monte Rosa nappe: **a** layered gneiss with quartz and biotite rich bands, and younger cross-cutting aplitic dyke. **b** Metamorphic assemblages: (i) large Grt formed during high temperature-low P Variscan metamorphism, and (ii) Wm + Ctd + St indicating peak conditions during the Alpine high P event. **c** Migmatitic textures of basement lithologies in close proximity to granitic intrusion indicating partial melting due to the intrusion of the Monte Rosa granite. **d** Wm + Ctd + St pseudomorphs after former contact metamorphic andalusite formed during the peak Alpine high P event. **e** Complexly folded mafic boudins in a matrix of coarse-grained Plg + Qz volcanoclastic metasediments belonging to the Furgg zone series. **f** Metamorphic assemblages: (i) large Grt formed during Variscan regional metamorphism, and (ii) Wm + Ctd + St belonging to peak Alpine high P event

The paragneisses of the Monte Rosa nappe exhibit a long metamorphic history. These pre-Variscan metasediments consist of complex layered gneisses of predominantly alternating garnet-biotite and quartz-plagioclase rich layers (Fig. 3a). Areas less affected by Alpine tectonic activity, in particular the eastern Perazzispétz ridge and the area south of Rifugio Guide d’Ayas (Fig. 2), preserve a relict assemblage of sillimanite (mostly replaced by kyanite), pinnitized cordierite, biotite and garnet + quartz, K-feldspar, muscovite and plagioclase (Bearth, 1952; Dal Piaz & Lombardo, 1986). This assemblage is related to a Variscan metamorphic event (Bearth, 1952, 1958; Dal Piaz & Lombardo, 1986; Engi et al., 2001; Vaughan-Hammon et al., 2021). Petrological investigations of these assemblages reveal a high-T metamorphic event related to an upper amphibolite facies sillimanite-K-feldspar metamorphism (ca. 700 °C/ 0.3–0.6 GPa; (Ferrando et al., 2002) associated with the Variscan orogenesis dated at ca. 330 Ma (Engi et al., 2001). Figure 3b shows large relict garnets, associated with pre-intrusive high-T Variscan metamorphism.

Migmatite textures are encountered throughout the polymetamorphic Monte Rosa basement (Fig. 3c). These migmatites occur near the post-Variscan intrusion of the Monte Rosa granite during the Permian, visible within areas that have experienced little to no Alpine overprinting deformation or metamorphism. We associate partial melting in this area of the nappe with intrusion of the Permian-age granitic body (269 ± 4 Ma; (Pawlig, 2001). The contact mineral assemblage is spatially associated with the Monte Rosa intrusive bodies. Areas less affected by deformation post-dating granitic intrusion (i.e. Perazzispétz ridge and the area south of Rifugio Guide d’Ayas, Fig. 2), exhibit textures of large statically grown minerals that have later been pseudomorphed (Fig. 3d). Recent work by Vaughan-Hammon et al. (2021) demonstrates that these pseudomorphs contain chloritoid, white mica and staurolite. This assemblage likely represents a high-pressure Alpine overprint of former contact metamorphic andalusite (Fig. 3d). North of the Rifugio Mezzalama, the metasediments in contact with the Permian granite are transformed into hornfels, and in some places, coarse-grained tourmaline is observed in former hornfels.

Associated with the Monte Rosa Variscan paragneisses are structural levels that contain diverse lithologies exhibiting a complex deformation history. These lithologies are typical for the so-called Furgg zone that mainly represents here a high-strain zone following the contact of the Monte Rosa nappe with the overlying ophiolites (Bearth, 1952, 1958; Jaboyedoff et al., 1996; Keller & Schmid, 2001; Steck et al., 2015). However, the exact definition of the Furgg zone (being part of the Monte Rosa nappe or an autochthonous cover of the Monte Rosa nappe) is disputed. The Furgg zone contains Paleozoic mafic boudins (Dal Liati et al., 2001; Luraschi, 2016; Piaz, 1966), and Permian-age leucocratic albite-rich layers (270.3 ± 1.5 Ma and 257 ± 3.5 Ma, (Luraschi, 2016), as well as younger mafic boudins that locally intrude Mesozoic metasediments, associated with volcano-sedimentary deposits (Jaboyedoff et al., 1996, their Fig. 5), and whose geochemistry differs from that of the neighboring Piedmont ophiolites (Kramer et al., 2003). We do not undertake detailed petrological work on these mafic boudins in order to distinguish different generations. For clarity, we have subdivided Furgg zone lithologies in the map into three sub-units: (1) garnet metapelites containing mafic boudins, (2) volcano-sedimentary layers containing mafic boudins (Fig. 3e), and (3) metacarbonates (Fig. 2). Particularly, north of the Rifugio Mezzalama and sporadically throughout the entire Monte Rosa nappe, outcrops of volcano-sedimentary horizons containing coarse grained plagioclase and quartz are observed. These are complexly folded with amphibolite-rich boudins (Fig. 3e). North of the Rifugio Mezzalama, lenses and elongated bodies of metacarbonates consisting of calcite, diopside, garnet and pseudomorphs after wollastonite, as well as quartz rich meta-arkose are observed. These metacarbonates occur as extremely long and thin horizons or as boudins due to subsequent tectonic reworking (Fig. 2). In this study, we do not elaborate further on the Furgg zone. We separate the Monte Rosa paragneisses from the Furgg zone based on outcrop investigation and the lithological criteria mentioned above (garnet metapelites with mafic boudins, volcano-sedimentary deposits with mafic boudins, and metacarbonates). Our interpreted geological history (Sect. 5.3) is based on that proposed in previous studies (e.g. Dal Piaz, 2001; Dal Piaz & Ernst, 1978; Dessimoz, 2005; Giacomazzi, 2017; Jaboyedoff et al., 1996; Keller & Schmid, 2001; Kramer, 2002; Steck et al., 2015).

Indicators of high-pressure metamorphism related to Alpine orogenesis are found throughout the Monte Rosa nappe. These consist of assemblages of phengite + paragonite + chloritoid + staurolite + garnet + kyanite + quartz in metapelites (1.6 ± 0.2 GPa and 585 ± 20 °C; (Vaughan-Hammon et al., 2021); Fig. 3f), and omphacite, glaucophane, garnet, phengite, lawsonite, rutile, and quartz in metabasic boudins of the Furgg zone that have not been subsequently retrogressed at amphibolite grade [1.3–2.7 GPa and 535–620 °C, (Ferrando et al., 2002; Gasco et al., 2011) Fig. 3e]. However, preserved eclogitic metabasic boudins are extremely uncommon in the mapped area.

Chlorite bearing domains are also common within the paragneiss basement, and are predominantly confined to post-peak, late Alpine shear zones (more descriptions within Sect. 3). Late Alpine brittle fractures are typically infilled with albite and/or quartz.

#### Permian metagranite intrusive suite

**Fig. 4:**
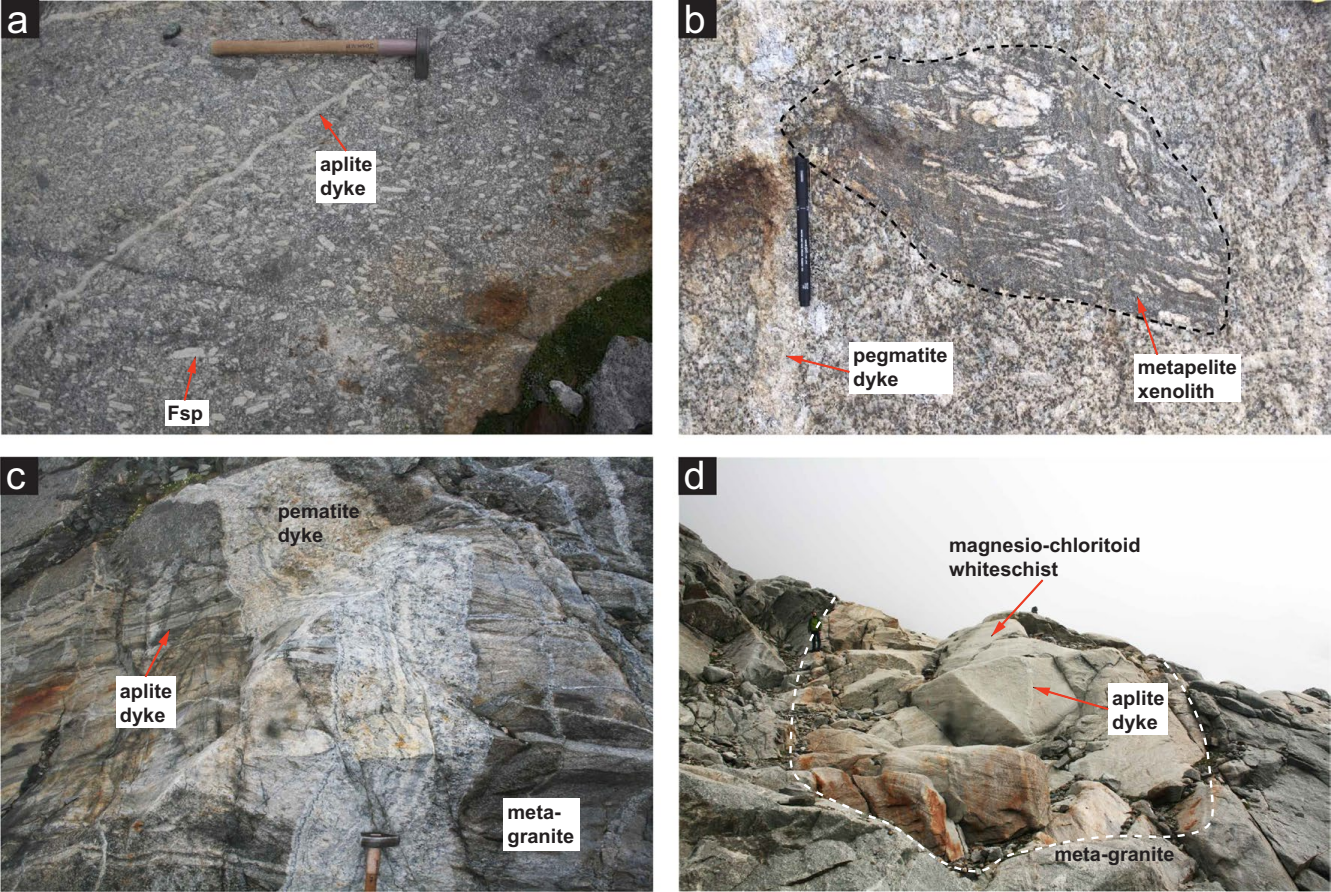
Mesoscopic aspect of key examples regarding intrusive contacts and structures belonging to the Monte Rosa nappe: **a** undeformed metagranite exhibiting magmatic textures with large K-feldspar phenocrysts, as well as a younger cross-cutting aplitic dyke. **b** Xenolith of Monte Rosa metapelitic Variscan basement exhibiting complex folding, enclosed within the metagranite. **c** Cross-cutting relationships of late magmatic dykes within Monte Rosa metagranite and metapelite lithologies (pegmatites are usually the youngest intrusive bodies). **d** Whiteschist indicating peak Alpine metamorphism enclosed within Monte Rosa metagranite lithologies (note aplitic dykes cross-cutting body- attesting to a granitic protolith)

In areas of low strain, the original intrusive porphyritic granite as well as leucocratic regions can be observed, consisting of coarse grained (> 10 cm) K-feldspar phenocrysts, quartz, biotite, plagioclase and muscovite (Fig. 4a). Remnants of basement paragneiss can be observed as xenoliths within the granite, structures within these xenoliths indicate a younger complex deformation history prior to Permian intrusion (Fig. 4b). Granodioritic enclaves are also observed throughout the granitic complex. Associated with the granite intrusion are a series of younger leucocratic dykes, cross-cutting both the granite and basement metapelites (Fig. 4a, c). Aplitic dykes most commonly consist of medium grained quartz, feldspar ± tourmaline. Coarse grained pegmatitic dykes are less common and consists of K-feldspar, white mica, quartz, tourmaline ± plagioclase ± garnet (Fig. 4a, c).

Alpine high-pressure relicts can be observed in metagranite lithologies, consisting of a fine-grained assemblage of zoisite + albite ± phengite, pseudomorphosing igneous plagioclase at ca. 1.4 GPa and 540–600 °C (Luisier et al., 2019). Unique assemblages of chloritoid, talc, phengite and quartz ± garnet, related to Alpine high P metamorphism, are observed in whiteschists (Chopin & Monié, 1984; Luisier et al., 2019; Pawlig & Baumgartner, 2001). These unique assemblages are exposed towards the north and south-east of the Rifugio Mezzalama, and it is here, in the grand cirque du Véraz, where we find the largest concentrations of these whiteschists in the Monte Rosa nappe (Figs. 2 and 4d). The whiteschists are either fully embedded in the metagranite (Fig. 4d), or appear as thin and elongated zones at the contact between Monte Rosa metagranite and basement paragneisses (Fig. 2). These whiteschists have been the focus of many studies characterizing their provenance, age and extent of metamorphic transformation (Chopin & Monié, 1984; Le Bayon et al., 2006; Marger et al., 2019; Pawlig, 2001). Extensive textural and chemical analysis of these whiteschist bodies through a shear zone reveal a late magmatic, hydrothermal metasomatic (Mg-enriched), chlorite-sericite schist protolith (Luisier et al., 2019, 2021; Marger et al., 2019; Pawlig & Baumgartner, 2001). Whiteschist lithologies show zoning patterns related to initial metasomatic alteration and subsequent retrogression after peak Alpine conditions; the studies of Luisier et al., (2021) and Pawlig and Baumgartner (2001) expand more upon this. The peak metamorphic conditions obtained from these whiteschist bodies during Alpine orogenesis has been recently estimated at 2.2 ± 0.2 GPa at 540–600 °C (Luisier et al., 2019).

## Structural observations

The Monte Rosa nappe records a complex structural history. Here we discuss 4 main deformation phases observed at the cirque du Véraz (S1 + F1, S2 + F2 + X2, S3 + F4 + X3, and S4 + B1), whereby S*(n)* corresponds to the resulting planar fabric such as schistosity, X*(n)* corresponds to a lineation, F*(n)* corresponds to a folding phase, and B*(n)* is a brittle planar fabric. All deformation phases except D1 (pre-intrusion of Permian granites) are related to Alpine orogenesis. Representative orientations and distributions of these structures are presented in the geological map of Fig. 2. We will discuss and compare these structures with previous studies and regional observations in Sect. 5.3 (Keller & Schmid, 2001; Keller et al., 2004; Kramer, 2002; Pawlig & Baumgartner, 2001; Steck et al., 2015).

**Fig. 5:**
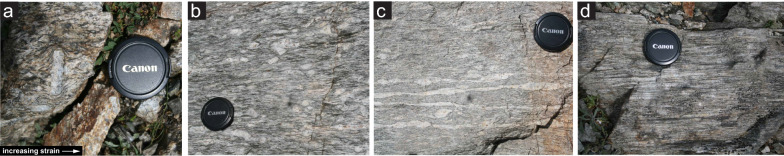
Compilation of field images illustrating the strain gradients in metagranitic lithologies of the Monte Rosa nappe: **a** weakly deformed porphyritic Monte Rosa metagranite showing biotite wrapping around a large partially recrystallized K-feldspar phenocryst. **b** Augen gneissic texture of K-feldspar phenocrysts and weakly interconnected biotite schistosity. **c** Formation of banded gneissic texture with highly strained and connected K-feldspar phenocrysts. **d** Proto-mylonitic texture with highly strained K-feldspar horizons, presumably former phenocrysts

A wide range of deformation intensities can be observed within the Monte Rosa metagranite and Variscan basement of the Monte Rosa nappe. These are best observed within the metagranite due to a spatially homogenous lithology and a presumably similar rheology, compared to the lithologically diverse and thus rheologically complex paragneiss basement. Figure 5 demonstrates the progressive increase in strain within the metagranites from: (a) weakly deformed porphyritic texture (original feldspar observed) to (b) augengneiss to (c) banded gneiss, and to (d) proto- and mylonite textures.

### Pre-intrusive ductile deformation: D1

**Fig. 6:**
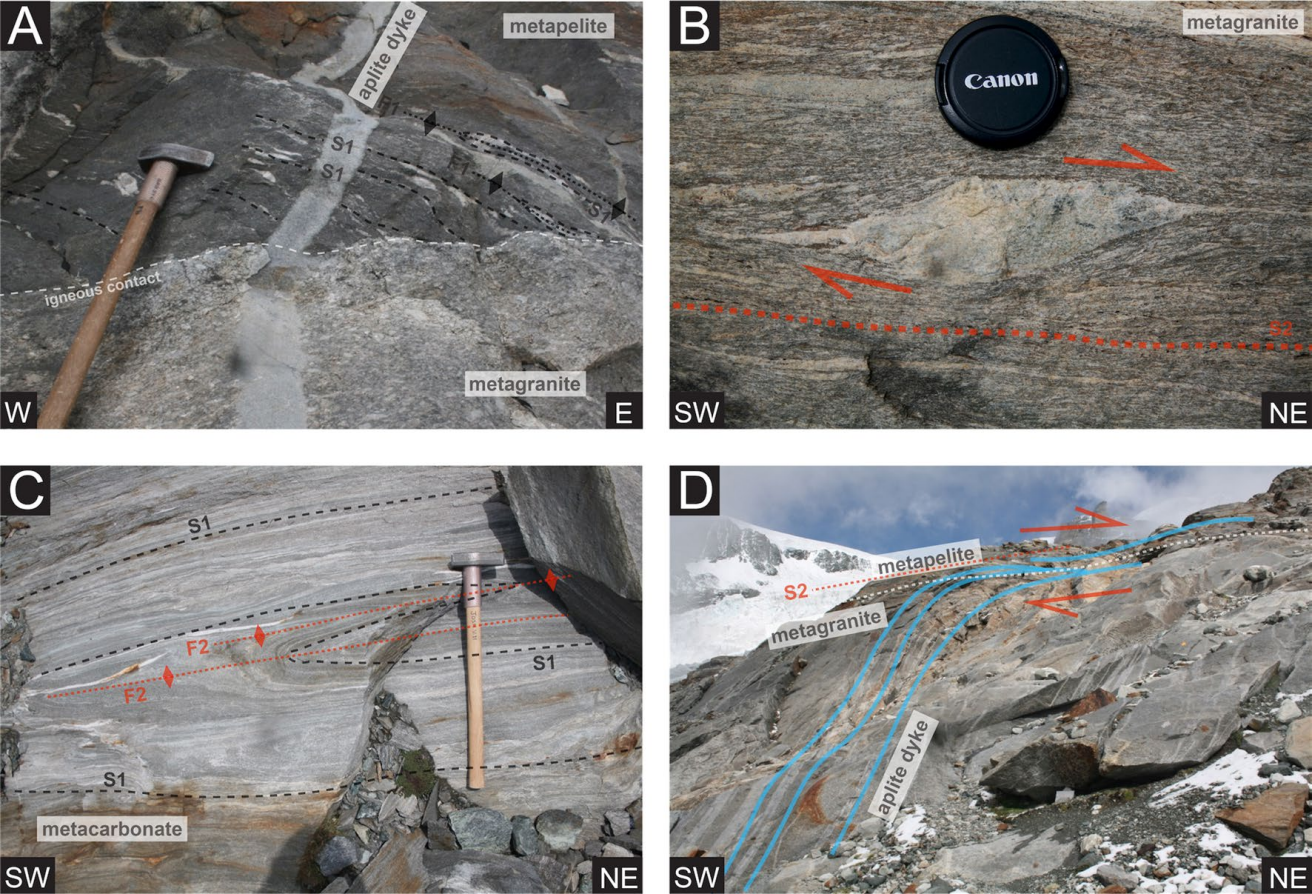
Representative field images of structural features in the Monte Rosa nappe: **a** magmatic contact between metagranitic and metapelitic lithologies preserving pre-intrusive Variscan structures such as schistosity (S1) and refolded isoclinal folding (F1). **b** Deformed aplitic dyke within Monte Rosa metagranite lithologies preserving S2 schistosity and dextral, top-N shear sense. **c** Isoclinally folded S1 schistosity of metacarbonate lithologies within the Monte Rosa basement with a dextral, top-N shear sense. **d** Large scale parallelization of aplitic dykes from undeformed Monte Rosa metagranite into rheologically weaker metapelites with a dextral, top-N shear related to F2 folding

The most ideal areas within the paragneiss basement to observe the oldest structures are areas that preserve an intact igneous contact, with minimal post-intrusion deformation (northeast of Rifugio Mezzalama and Perazzispétz, Fig. 2). These areas enable constraints on the relative ages of deformation, in order to distinguish pre-intrusive basement structures. Figure 6a shows a well preserved original igneous contact marked with a late aplitic dyke, demonstrating the complex deformation already present prior to the Permian-aged granitic intrusion. Superposition of several complex phases of folding can be observed in refolded isoclinal quartz veins (Fig. 6a). S1 and F1 define phases of schistosity development and folding events that coincide with pre-intrusive tectonic activity. We do not elaborate further on these early pre-intrusion structures of pre-Alpine age since this is not the focus of this study.

The Furgg zone (metacarbonates, metamafics and metavolcanoclastics) displays a complex high strain history (Figs. 3e and 6c). Extensive Alpine tectonic reworking has resulted in complex folding with the Monte Rosa paragneiss (particularly north of the Rifugio Mezzalama Fig. 2).

### Ductile deformation: D2

**Fig. 7:**
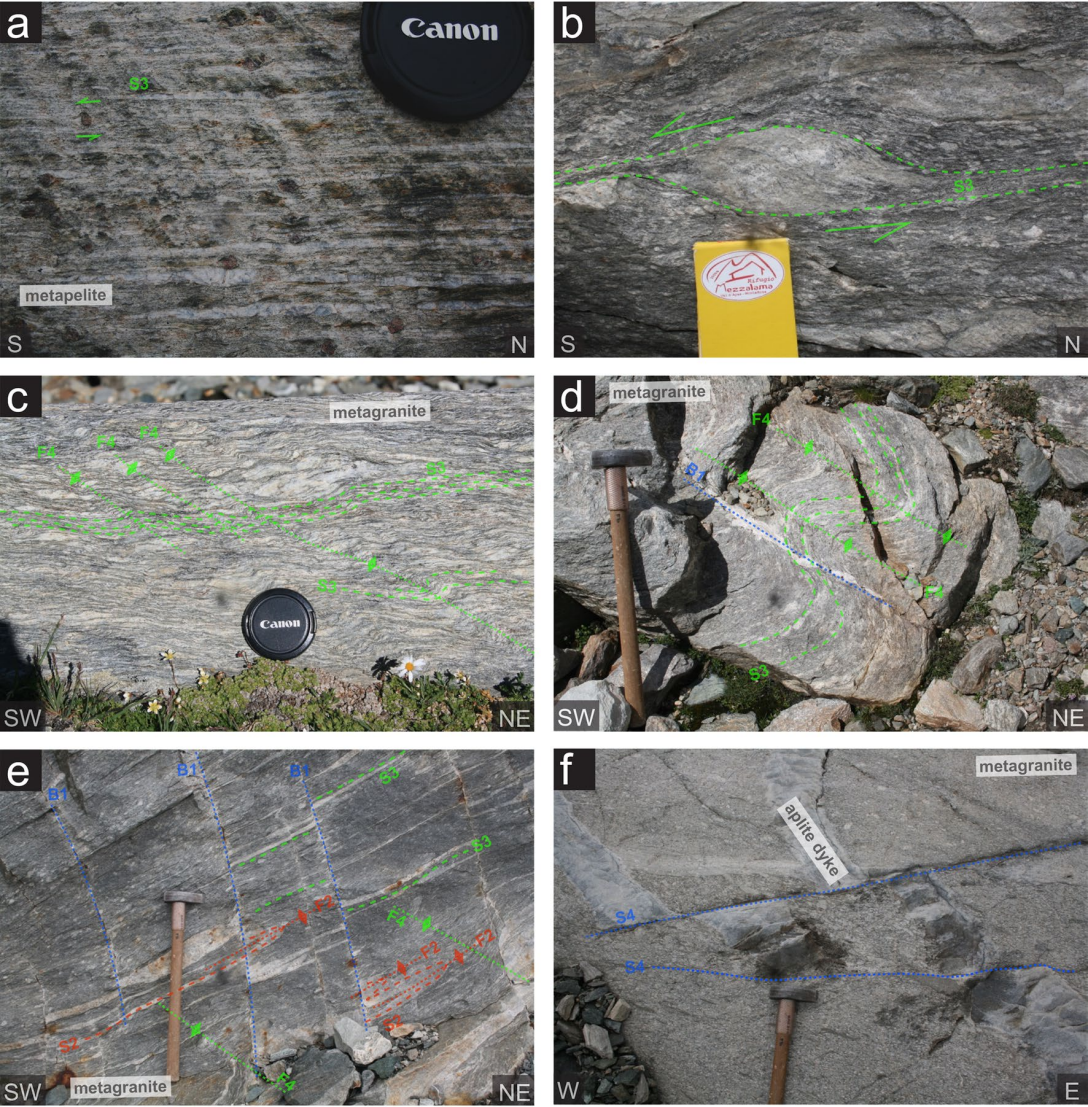
Representative field images of structural features belonging to the Monte Rosa nappe: **a** Highly strained schistosity (S3) of deformed garnet-chlorite bearing metapelites, preserving sinistral, top-SW shear sense. **b** Deformed aplitic dyke within Monte Rosa metagranite lithologies preserving S3 schistosity and sinistral, top-SW shear sense. **c** S3 schistosity in metagranite and weak asymmetrical crenulation related to F4 folding with a sinistral, top-SW shear sense. **d** F4 folding of S3 schistosity in metagranite with cross-cutting S4 brittle fracture that is subsequently filled with albite. **e** highly deformed metagranite with 3 generations of cross-cutting deformation structures. **f** Example of brittle S4 features offsetting aplitic dykes within the metagranite

**Fig. 8:**
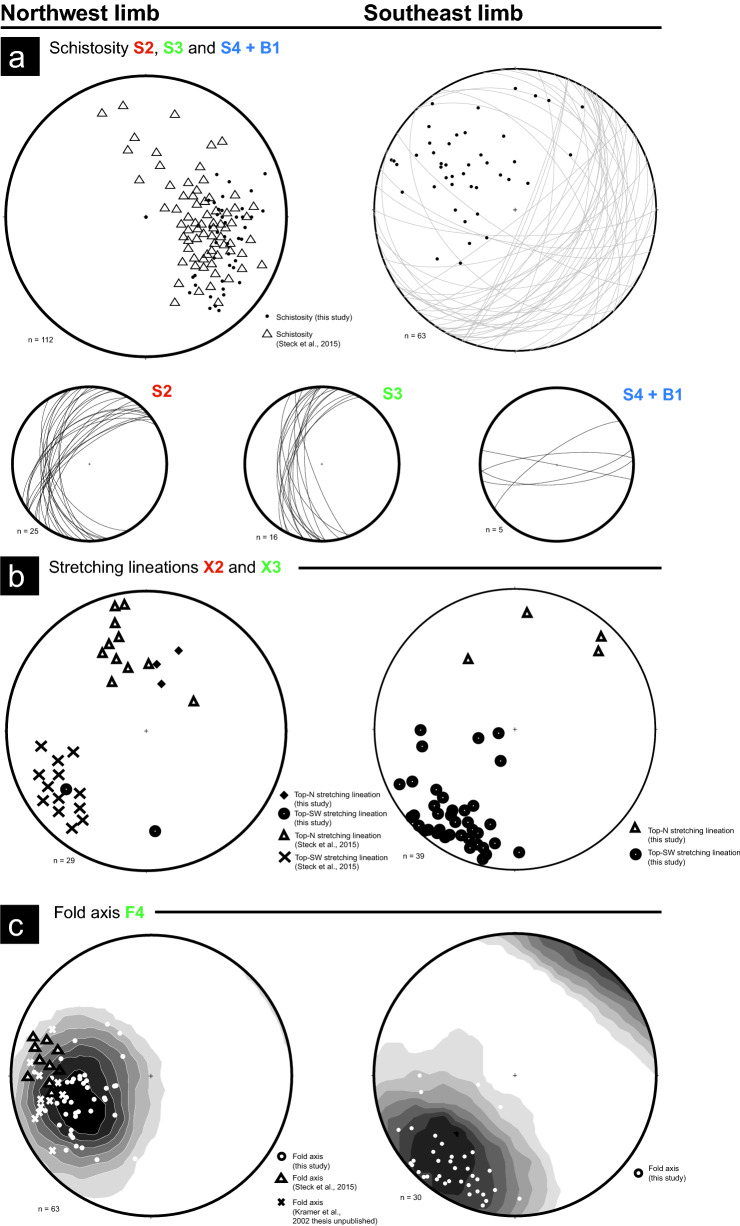
Lower hemisphere stereographic projections of major structural features of the Monte Rosa nappe [including former studies in cirque du Véraz area Kramer, 2002; Steck et al., 2015)], taken from the northern and southern limb of the larger F4 fold (Fig. 2 cross-section)

**Fig. 9:**
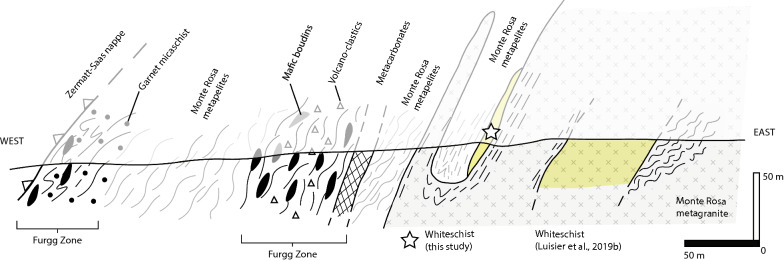
Schematic E–W lithological section from the Monte Rosa metagranite intrusive suite, Monte Rosa basement complex, and finally the over-thrusted Zermatt-Saas nappe. Structural position of whiteschist analyzed in Luisier et al. (2019) and the whiteschist of this study are indicated

**Fig. 10:**
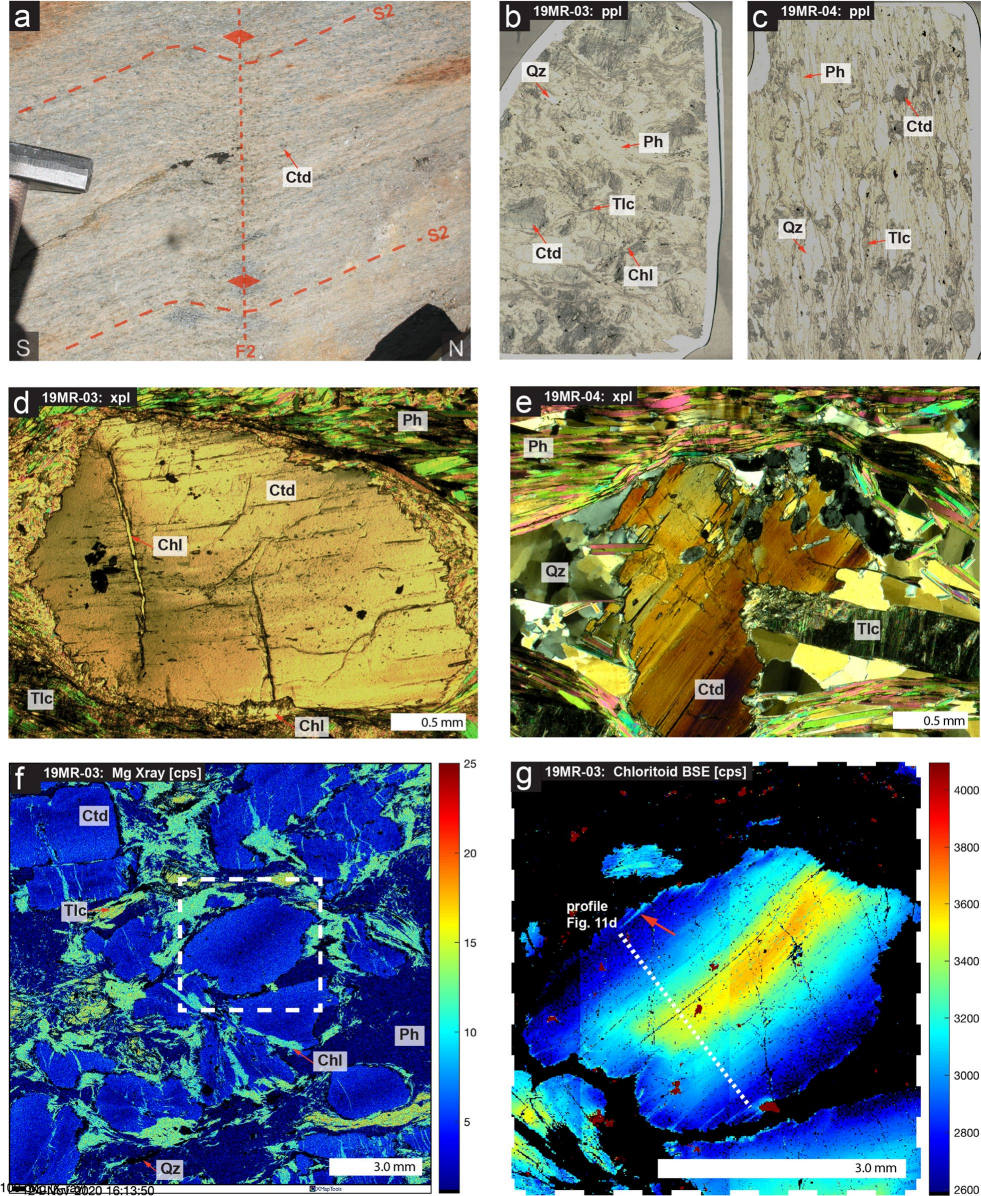
Petrological features of whiteschist samples 19MR-03 and 19MR-04: **a** field image of S2 schistosity and subsequent F2 dextral, top-N minor crenulation. **b** whole thin-section plane polarized light image of coarse-grained chloritoid whiteschist sample 19MR-03. **c** whole thin-section plane polarized light image of fine-grained whiteschist sample 19MR-04. **d** photomicrograph in cross-polarized light of late chlorite growth within coarse grained chloritoid fractures. **e** photomicrograph in cross-polarized light of typical textures observed in fine grained whiteschist samples 19MR-04. **f** X-ray map of Mg in sample 19MR-03, note high Mg talc and visible zoning in large chloritoid grains. **g** major zoning observed in BSE image for chloritoid grains, profile represented for microprobe analysis (Fig. 11d) [XMapTools Lanari et al. (2014)]

The first phase of post-intrusion deformation is predominantly seen as ductile S2 shear zones and affects both the basement complex and the metagranite (including whiteschists, Figs. 10a, 6b, c). This deformation is typically associated with a ductile foliation indicating a top-N sense of shear and northward directed lineation X2 (Figs. 6b, c, 8a and 9a). North of the Rifugio Mezzalama, we can observe top-N shearing of aplitic dykes that remained undeformed in the metagranite and become progressively sheared within the metapelite corresponding to drag into shear zones (Fig. 6d). Equally, increasing strain can produce isoclinal F2 folding of dykes, which is associated and parallel to S2 features (Figs. 6c and 7e).

The metamorphic characteristics of this deformation event typically post-date high P phases (deformed high P garnet + white mica in metapelites and metagranites, as well as whiteschists from this study). A greenschist overprint is present also, which is manifested as chlorite + epidote shear zones within the metagranite.

### Ductile deformation: D3

S3 schistosity within both metapelites and metagranites is associated with a top-S sense of shear (X3 lineations) and can be observed to re-activate former top-N, S2 fabrics, but with an opposite shear sense (Fig. 7a, b). F3 folding associated with S3 is not observed. However, by far the most pervasive and clear deformation event observed to overprint S3 is widespread folding F4 (Fig. 7c). F4 folding is ubiquitous and typically corresponds to a fold axial plunge dipping ~ SW and postdates previous schistosities (S1, S2 and S3) as most schistosities hinge around the pole of a ~ SW dipping F4 axis (Fig. 8c). F4 is characterized by an asymmetrical folding style with a top-S sense of shear (Fig. 7c), therefore we consider this deformation D3 closely associated and post-dating S2. This folding can be observed as minor to tight asymmetrical crenulations, drag folds and km-scale open folds (Fig. 2 cross-section). Figure 8c shows the extent of F4 folding whereby both S2 and S3 (Fig. 8a) are observed to rotate around the pole of the F4 axial plunge (Fig. 8c). Moreover, S2 has been influenced much more by this folding compared to S3 attesting to its relatively younger age. The large scale folding evident from the cross-section in Fig. 2 is seen in stereographic projections for S2 and S3 fabrics north and south of the hinge point (Fig. 8). North of the hinge point, S2 and S3 schistosity dips to the northwest whereas south of the hinge point schistosity dips to the southeast (Fig. 8a).

The metamorphic characteristics of this deformation event is a common greenschist overprint, manifested as chlorite + epidote shear zones throughout.

### Orogen-parallel ductile and brittle deformation: D4

A transition from ductile to brittle deformation marks one of the last phases of deformation in the area, S4 and B1 (Figs. 7d–f). Ductile features S4 are thin (1–5 cm) discrete shear zones with 1–5 m offset (Fig. 7f). Brittle features B1 are typically manifest as 1–5 cm wide fractures within the metagranite with minimal offset (Fig. 7d), and is associated with infillings of albite and/or quartz veins. Planar fabrics of S4 and B1 both typically have an E–W orogen-parallel strike (Fig. 8a).

## Additional outcrop of whiteschist

A new outcrop of whiteschist has been discovered due to recent snow melt, uncovering fresh exposure north of the Rifugio Mezzalama (E: 2624890 N: 1084985; see location of samples 19MR-03 and 19MR-04 in Fig. 2). This whiteschist is in close proximity to previous whiteschists investigated by Luisier et al. (2019). Figure 9 shows a schematic section of the Monte Rosa basement complex, Furgg zone and Zermatt-Saas ophiolites, indicating the position of the new whiteschist outcrop relatively to the already described whiteschist outcrops (Le Bayon et al., 2006; Luisier et al., 2021; Marger et al., 2019; Pawlig & Baumgartner, 2001). In contrast to the whiteschist investigated by Luisier et al. (2019), the newly discovered whiteschist is located at the contact between Monte Rosa metagranite and basement paragneiss. It comprises predominantly phengite, talc, quartz, chloritoid ± chlorite (Fig. 10). In some areas of the whiteschist, chloritoid reaches over 1 cm in size (Fig. 10a). We have sampled both coarse-grained (19MR-03, Fig. 10b) and fine-grained areas (19MR-04, Fig. 10c) for petrological analysis.

Particularly in sample 19MR-04, a schistosity is defined by deformed phengite, talc, quartz and chloritoid (048/58 NW) that is parallel to the metagranite and metapelite contact and lacks a chlorite overprint (sample 19MR-04, Fig. 10a, c, e). This schistosity, likely S2, is similar to deformation structures in surrounding lithologies, and shows a minor later kink fold (Fig. 10a). Since whiteschist lithologies reflect peak Alpine metamorphic conditions in the Monte Rosa nappe according to previous authors, this outcrop will enable us to constrain the nature of deformation after the peak Alpine high P metamorphism and before subsequent greenschist overprinting typical of S2 and S3 structures.

Metapelites from an outcrop in close proximity to these whiteschists exhibit a static, medium-P, albite-epidote amphibolite Alpine metamorphic assemblage (garnet, phengite, biotite, quartz), and a schistosity parallel to S2 that post-dates the peak Alpine paragenesis. Microstructural evidence (Sect. 4.2) suggests that this assemblage is then retrogressed and replaced with chlorite without significant deformation (Vaughan-Hammon et al. (2021).

### Methodology

Electron probe microanalysis (EPMA) of major and minor element compositions of white mica, chloritoid and chlorite were conducted using a JEOL JXA-8350F HyperProbe at the University of Lausanne, Switzerland. The operating conditions were 15.0 kV acceleration voltage and 1.5–2.0 × 10^–8^ A, with a beam diameter of 5.0 μm. Natural minerals were used as reference materials: orthopyroxene (SiO_2_), andalusite (Al_2_O_3_), albite (Na_2_O), fayalite (FeO), forsterite (MgO), orthoclase (K_2_O), nephrite (MnO), wollastonite (CaO), sphalerite (ZnO), rutile (TiO_2_). Structural formulae were calculated on the basis of 11 oxygens for white mica and talc, 8 cations for chloritoid and 13 oxygens for chlorite.

Phase diagrams were calculated using the DOMINO software (de Capitani & Brown, 1987) in combination with the Berman database (Berman, 1988, 92 update). This database was chosen in order to: (1) have an internally consistent database which is based nearly exclusively on experimental data, and (2) to accurately compare thermodynamically calculated P and T with the study of (Luisier et al., 2019), who also utilized this database to calculate metamorphic conditions for whiteschist assemblages. Bulk compositions were calculated based on quantitative image analysis of whole thin section domains in each sample, using EPMA derived mineral compositions, along with the MATLAB© based image processing software XMapTools (Lanari et al., 2014), in the chemical system KFMASH. Solution models used are from the 92 update of Berman (1988) data base (chloritoid and chlorite) and further included the white mica model after Massonne and Szpurka (1997). Mineral abbreviations are after Whitney and Evans (2010).

Talc is a major constituent of the whiteschist mineralogy. However, due to the only existing end-member solution data for talc being Mg (Berman, 1988, 92 update), it is necessary to account for the presence of other non-negligible talc end-members (e.g. Fe-talc) when calculating pseudo-sections. We have employed an entropy correction (${S}^{corr}$), defined here by ${S}^{corr} ={S}^{o}- Rln a$, where ${S}^{o}$ is the entropy for the pure phase. A full explanation and derivation of this correction is outlined in Appendix 1 of (Vaughan-Hammon et al., 2021). For a pure phase, $a$ = 1 and ${S}^{corr} ={S}^{o}$, thus the entropy within the JUN92B database by Berman (1988) is unaffected. The existing solution model for talc is for Mg-rich end-members. Here we adjust the activity ${a}_{tlc}^{Mg}$ of the existing Mg-talc in the database employing a site mixing model: $a= {a}_{tlc}^{Mg} ={{X}_{Mg}}^{3}$, where X_Mg_ = Mg/(Mg + Fe).

### Petrography

Sample 19MR-03 displays a coarse-grained texture of 0.5–1 cm sized chloritoid in a finer grained matrix (< 1 mm) of predominantly weakly orientated phengite, as well as talc and quartz (Fig. 10b). The large chloritoids display fracture networks that form subgrains in some places and are associated with late chlorite precipitation (Fig. 10d, f). Areas rich in talc within the matrix seldom form interconnected textures, rather isolated regions. Estimates of modal volumes of minerals include: 33% phengite, 36% chloritoid, 7% quartz, 14% chlorite, and 9% talc.

Sample 19MR-04 displays a finer grained texture compared to 19MR-03, consisting of ~ 2 mm sized chloritoids (Fig. 10c, e). This sample has a much more developed schistosity defined by fine grained phengite and talc as well as having a larger volume of quartz, defined as regions of elongated recrystallized quartz aggregates (Fig. 10e). Phengite and talc are observed to wrap around rigid chloritoid grains, with quartz (and minor amounts of talc and phengite) precipitating within strain shadows (Fig. 10e). No syn-kinematic growth of minerals is observed. Kink bands and foliation of white mica, deformation of all mineral phases, lack of preserved foliations within chloritoid and the general lack of syn-kinematic growth indicators of chloritoid suggests pre-tectonic (pre-deformation) growth of the whiteschists. No chlorite is observed in sample 19MR-04. Estimates of modal volumes of minerals include: 27% phengite, 16% chloritoid, 47% quartz, and 10% talc.

### Mineral chemistry and pseudo-section results

Representative microprobe results for whiteschist samples 19MR-03 and 19MR-04 are presented in Table 1. The peak paragenesis for both samples consists of chloritoid (Ctd), phengite (Ph), talc (Tlc) and quartz (Qz).

**Table 1:** Representative microprobe analysis (19MR-03 and 19MR-04)

Analysis	19MR-03					19MR-04		
	Phengite	Talc	Chloritoid		Chlorite	Phengite	Talc	Chloritoid
			Core	Rim				
SiO_2_	52.60	62.59	24.33	25.14	26.46	52.37	62.76	25.68
Al_2_O_3_	27.42	0.22	43.14	44.73	23.27	27.87	0.19	43.93
TiO_2_	0.26	0.02	0.00	0.01	0.07	0.23	0.01	0.00
MnO	0.01	0.00	0.34	0.11	0.06	0.00	0.00	0.09
ZnO	0.01	0.00	0.00	0.01	0.03	0.00	0.01	0.04
FeO	0.76	3.06	20.66	13.38	12.96	0.93	3.28	13.86
MgO	4.27	30.17	5.43	9.87	24.70	4.15	29.52	9.15
Na_2_O	0.31	0.02	0.01	0.00	0.01	0.35	0.02	0.00
CaO	0.00	0.00	0.01	0.00	0.02	0.00	0.03	0.01
K_2_O	11.1	0.00	0.01	0.00	0.03	10.83	0.01	0.00
Total	96.7	96.09	93.70	93.25	87.63	96.73	95.82	92.76

Normalized^a^	11 (a)	11 (a)	8 (c)	8 (c)	13 (a)	11 (a)	11 (a)	8 (c)
Si^4+^	3.435	3.976	1.930	1.937	2.494	3.417	3.998	1.999
Al^3+^	2.111	0.017	4.032	4.060	2.491	2.143	0.014	4.029
Ti^4+^	0.013	0.001	0.001	0.000	0.005	0.011	0.000	0.000
Mn^2+^	0.001	0.000	0.023	0.007	0.004	0.000	0.000	0.006
Zn^2+^	0.001	0.000	0.000	0.000	0.002	0.000	0.001	0.002
Fe^2+^	0.042	0.163	1.370	0.862	0.910	0.051	0.175	0.902
Mg^2+^	0.416	2.857	0.642	1.133	3.344	0.404	2.804	1.062
Na^+^	0.040	0.002	0.002	0.000	0.002	0.045	0.002	0.001
Ca^2+^	0.000	0.000	0.001	0.000	0.002	0.000	0.002	0.001
K^+^	0.921	0.000	0.001	0.000	0.004	0.902	0.001	0.000
Total	6.980	7.016	8.002	7.999	9.258	6.973	6.997	8.002

^a^Normalization using anions (a) and cations (c)

**Fig. 11:**
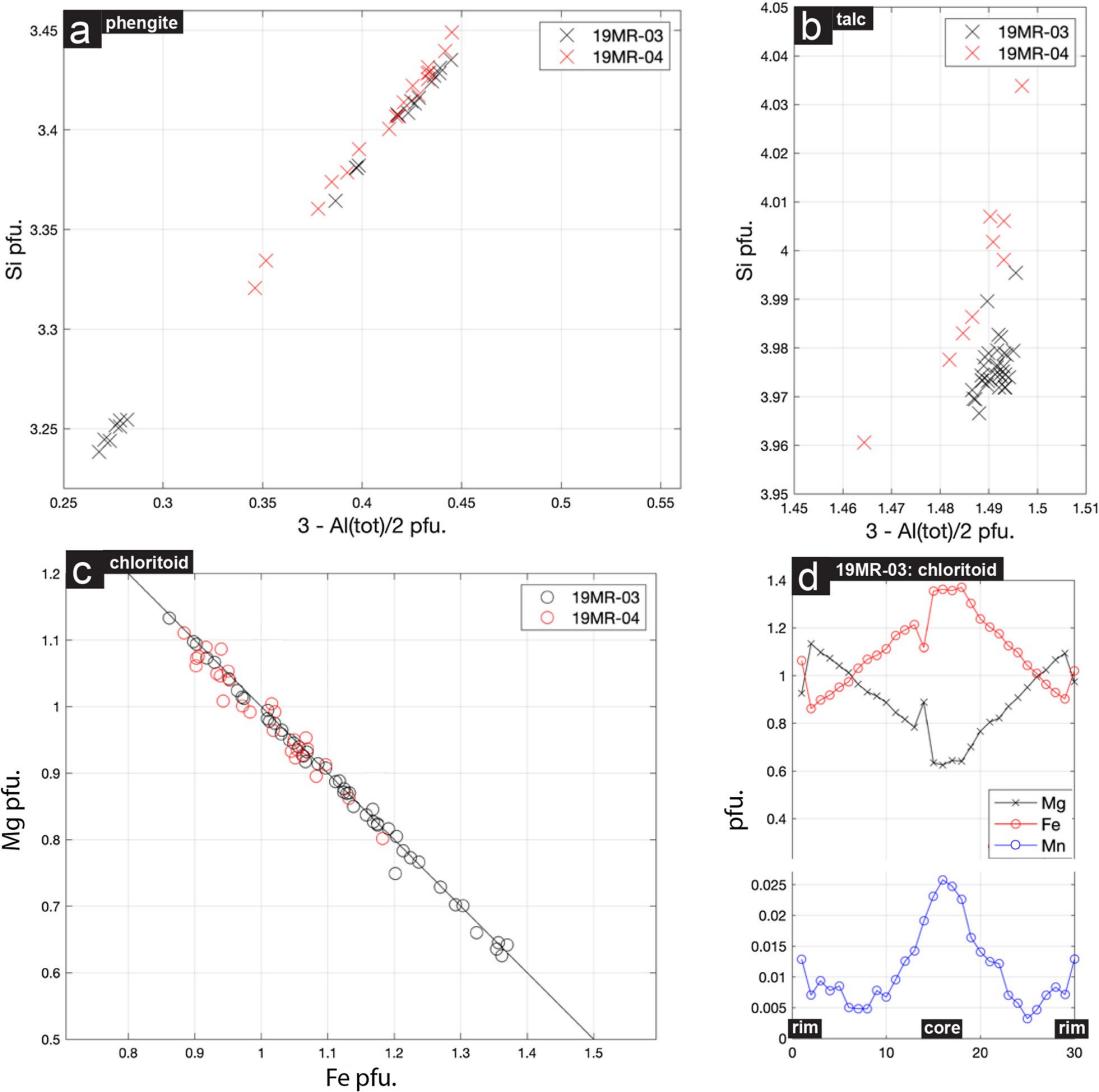
Normalized mineral chemical data for whiteschist assemblages in samples 19MR-03 and 19MR-04: **a** Si in phengites against Al tetrahedral sites. **b** Si in talc against Al tetrahedral sites. **c** Mg vs Fe-total in chloritoid. **d** Representative zoning of Mg, Fe and Mn in coarse-grained chloritoid of sample 19MR-03

Within 19MR-03, coarse grained chloritoid shows significant zoning (Figs. 10f, g and 11d). X_Mg_ (Mg/(Mg + Fe)) is smallest within the core of chloritoid grains at 0.32. Towards the rims of chloritoid, X_Mg_ increases to 0.57. Equally so, minor zoning of Mn can be observed from core to rim, at 0.008 to 0.025 atoms per formula unit (a.p.f.u) (Fig. 11d). Phengites show a broad range of Si content ranging between 3.23 and 3.43 a.p.f.u from several different generations of white mica, reflecting a range of the Tschermak component between 0.26 and 0.44 (Fig. 11a). Phengites exhibit a moderate range of X_Na_ ((Na + Na)/K) between 0.10 and 0.21. Talc shows a rather narrow range of Si content ranging between 3.96 and 4.0 a.p.f.u, corresponding to a Tschermak component between 1.48 and 1.5 (Fig. 11b). Talc is enriched in Mg (Table 1) with a narrow range of X_Mg_ between 0.93 and 0.95. Late stage chlorite is chemically nearly homogenous throughout the sample with an X_Mg_ of 0.80.

**Fig. 12:**
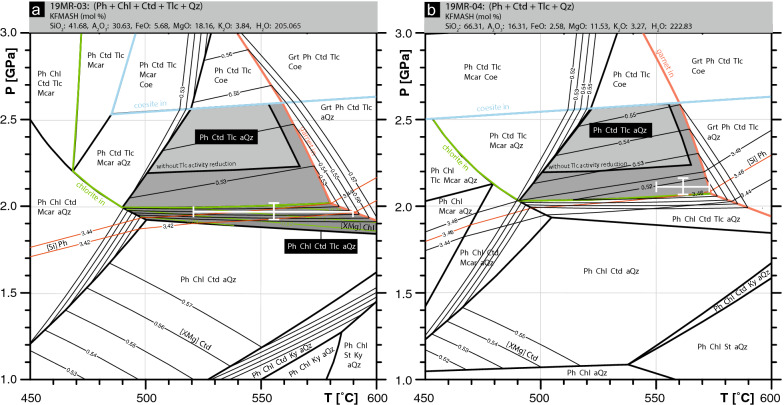
Thermodynamic pseudo-section modelling for sample 19MR-03 and 19MR-04. **a** KFMASH pseudo-section calculated for sample 19MR-03, inferred peak assemblage indicated as Ph + Ctd + Tlc + Qz as well as retrogressional assemblage stability field of Ph + Ctd + Tlc + Qz + Chl. Enlarged stability field of peak assemblage resulting from the talc activity reduction due to Mg- and Fe-talc end-members. Si in phengite isopleths, X_Mg_ in chloritoid (Mg/(Mg + Fe)) isopleths, and X_Mg_ in chlorite isopleths. **b** KFMASH pseudo-section calculated for sample 19MR-04, peak assemblage indicated as Ph + Ctd + Tlc + Qz as well as the enlarged stability field resulting from the talc activity reduction due to Mg- and Fe-talc end-members. Si in phengite isopleths and X_Mg_ in chloritoid are present

To gain insight into the phase petrology of these samples, we first calculated pseudo-section or metamorphic assemblage diagrams (MAD, Spear & Pattison, 2017). Figure 12a shows the calculated pseudo-section results for 19MR-03. For the peak assemblage of Ph + Ctd + Tlc + Qz, a large stability field is calculated. Employing the activity correction outlined in the methods section, the resulting stability field is enlarged towards lower pressures, indicated in the pseudo-section of Fig. 12a. Due to the presence of late chlorite (Chl), the retrograde path is indicated by the chlorite-in reaction of Ph + Chl + Ctd + Tlc + Qz (Fig. 12a). This stability field agrees with bulk chemistry isopleths of chloritoid X_Mg_, Si in phengite, and chlorite X_Mg_ (Fig. 12a). The resulting metamorphic conditions for Ph + Chl + Ctd + Tlc + Qz taking into account errors is 1.95 ± 0.05 GPa and 555 ± 35 °C.

Sample 19MR-04 has minimal zoning in finer grained chloritoids with X_Mg_ of 0.49 (Table 1). Phengites show a narrow range of Si content ranging between 3.32 and 3.45 a.p.f.u, which correlates with an increase of the Tschermak component between 0.34 and 0.44 (Fig. 11a). Phengites exhibit a moderate range of X_Na_ between 0.10 and 0.16 which are relatively lower values compared to 19MR-03. Talc shows a range of Si content between 3.96 and 4.04 a.p.f.u, which correlates with weak (if not absent) Tschermak component between 1.46 and 1.5 (Fig. 11b). Talc is enriched in Mg (Table 1) with a narrow range of X_Mg_ between 0.89 and 0.91.

Figure 12b shows the calculated pseudo-section results for 19MR-04. The peak assemblage of Ph + Ctd + Tlc + Qz is represented with and without the Mg-talc entropy correction. Isopleths for measured chloritoid X_Mg_ and Si in phengite are represented for the Mg-talc entropy correction stability field (Fig. 12b). The isopleths cluster towards higher temperatures and lower pressures within the enlarged stability field. The resulting metamorphic conditions for Ph + Ctd + Tlc + Qz taking into account errors is ca. 2.1 ± 0.2 GPa and 560 ± 20 °C.

## Discussion

### Whiteschist results

#### Comparison with previous studies

The results for peak Alpine metamorphic conditions incurred by whiteschist lithologies in this study have been calculated at 2.1 ± 0.2 GPa and 560 ± 20 °C (Fig. 12b). This result agrees with the estimated peak conditions of ca. 2.2 ± 0.2 GPa at 540–600 °C for whiteschist assemblages analyzed by Luisier et al. (2019), taken from a nearby outcrop (Fig. 2). Le Bayon et al. (2006) also sampled a whiteschist from the nearby whiteschist outcrop and calculated peak conditions at 2.4 ± 0.2 GPa and 500 ± 30 °C. Le Bayon et al. (2006) use an averaged bulk composition in their study and a H_2_O activity between 0.59 and 0.66. They do not elaborate on the interpretation of the reduced activity; some initial calculations by us suggest that if the fluid activity were reduced due to the presence of CO_2_, then this would lead to the stability of magnesite under the proposed conditions, which is, however, not observed. Several decades ago, Chopin and Monié (1984) already analyzed whiteschist samples taken from a location close to the outcrop of this study (western side of the Véraz glacier, Fig. 2). However, it originated from a loose moraine block. A comparison of our mineral chemistry with that of Chopin and Monié (1984), as well as the position and altitude their sample was taken from, suggest that both their and our whiteschists may have originated from the recently uncovered outcrop (Fig. 2). However, our peak metamorphic conditions at 2.1 ± 0.2 GPa and 560 ± 20 °C do not agree with that of Chopin and Monié (1984) at ca. 1.6 GPa and 500 °C. This is likely due to a lowered H_2_O activity of 0.6 used in Chopin and Monié (1984), based on the presence of fluid inclusions with a gas bubble, which was not further studied. This lowered H_2_O activity resulted in a temperature of 500–550 °C (Chopin & Monié, 1984), suggested by the absence of Alpine staurolite in the Monte Rosa basement metapelites. A T below 550 °C allowed the Cretaceous Ar–Ar ages obtained to be interpreted as reflecting peak Alpine conditions. Chopin and Monié (1984) argue that a higher T would have resulted in a resetting of the ages during cooling. At this point, however, it is more probable that these ages are artifacts of inherited Ar, as is often observed in high-P rocks (e.g. Laurent et al., 2017; Pawlig, 2001), since high pressure metamorphism in the Monte Rosa has now been dated at roughly 40 Ma (Lapen et al., 2007). Recent geochemical analysis by Luisier et al. (2019) provides estimates of H_2_O activity being close to 1. Furthermore, a more recent study based on the discovery of locally occurring Alpine staurolite-bearing metapelites in the Monte Rosa basement indicates a higher peak Alpine T for the Monte Rosa nappe of < 600 °C (Vaughan-Hammon et al., 2021).

Pressure conditions of > 2.2 GPa for the whiteschist do not agree with numerous studies undertaken in other portions of the western Monte Rosa nappe for different lithologies including: metagranitic assemblages < 1.6 GPa (Luisier et al., 2019), metapelitic assemblages showing peak P of 1.6 ± 0.2 GPa and 585 ± 20 °C (Vaughan-Hammon et al., 2021) and 1.35 GPa and 670 °C (Keller et al., 2004), and metapelitic and metabasic assemblages showing 1.3 GPa and a peak T of 546 ± 21 °C (Borghi et al., 1996) and 1.4 GPa and 440–530 °C (Dal Piaz & Lombardo, 1986). A study on metabasic assemblages reports peak conditions of ca. 2.7 GPa and 570 °C (Gasco et al., 2011). However, P estimates obtained by Gasco et al. (2011) are debated due to insufficient mineral solid solution models (Lardeaux, 2014). Equally, these metabasic boudins likely derive from the Furgg zone. One suggestion for this disparity is the presence of a tectonic mélange.

The majority of whiteschist bodies studied in the cirque du Véraz area originate from outcrops that are enclosed within metagranite lithologies (Le Bayon et al., 2006; Luisier et al., 2019; Pawlig & Baumgartner, 2001). For the two whiteschist samples analyzed in this study, the situation is different, as they originate from a lensoidal body located directly at the contact between the Monte Rosa metagranite and basement complex (Figs. 2 and 9). This may pose difficulties when trying to attribute a protolith to the whiteschist (either metagranite or metapelite). However, the similarity in geochemistry to that of Luisier et al. (2019) agrees with the concept of a late hydrothermal metasomatic alteration (Mg-enrichment) of a granitic protolith that occurred prior to Alpine deformation (Luisier et al., 2019, 2021; Marger et al., 2019; Pawlig & Baumgartner, 2001). Moreover, no field evidence suggests tectonic mixing of whiteschist and metagranite or metapelite. Furthermore, all deformation exhibited within the whiteschist samples of this study represents post-peak P strain. We do not observe any indications of syn-deformational growth of high-pressure minerals in the whiteschist (talc, phengite, chloritoid + quartz). Specifically, in 19MR-04, textural observations indicate a post-crystallization deformation event (Fig. 10c, e). The lack of retrograde minerals suggest that the deformation was either soon after peak conditions, still within the stability field of the peak assemblage or alternatively, no fluids were available to form retrograde minerals in that sample. However, the coarser grained 19MR-03 has some late (retrograde) chlorite within brittle features of large chloritoid grains (19MR-03, Fig. 10b, d, f). A similar minor overprinting with chlorite is observed surrounding chloritoid, resulting in a zonation pattern that exhibit a decrease in Mg and an increase in Fe and Mn (Fig. 11d).

#### Modelling prograde chloritoid zoning

**Fig. 13:**
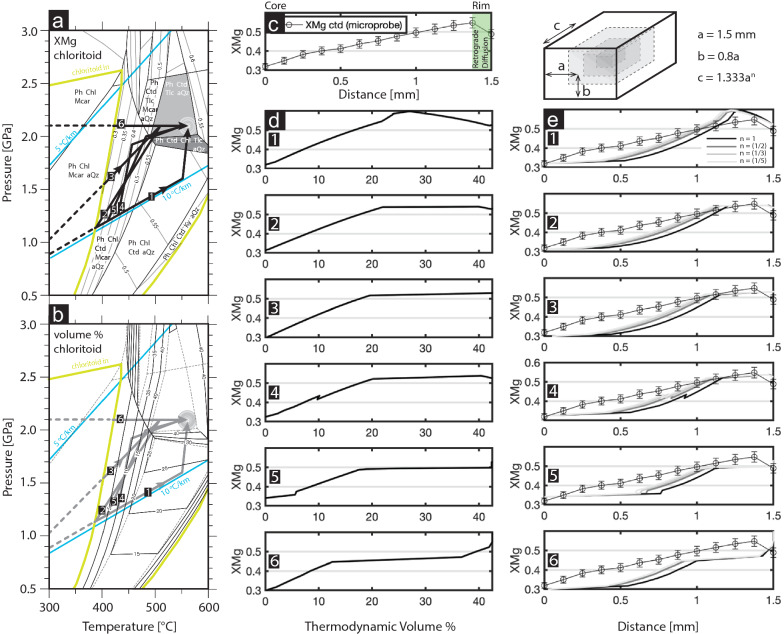
Comparison of measured X_Mg_ chloritoid zoning and thermodynamically calculated X_Mg_ chloritoid zoning: **a** Thermodynamic pseudo-section, identical to pseudo-section presented in Fig. 11a including X_Mg_ values for chloritoid. Prograde pathways 1–6 marked with black arrows. Geotherms for 5 and 10 °C/km marked with blue lines. **b** Thermodynamic pseud-section with volume% of chloritoid. **c** Measured microprobe X_Mg_ of chloritoid from core to rim of sample 19MR-03. **d** pseudo-section derived X_Mg_ of chloritoid for sample 19MR-03 for the various prograde pathways represented in **a** and **b**, plotted against pseudo-section predicted volume% of chloritoid. **e** pseudo-section derived X_Mg_ of chloritoid plotted against a radius calculated from the predicted volume% of chloritoid during prograde growth for a rectangular geometry (see Appendix 1 for details about exponential factor *n*)

Due to the variability in peak P incurred by the Monte Rosa nappe during Alpine orogenesis, the P–T path during burial and exhumation is still contentious. In order to assess the possible P–T paths during prograde metamorphism, we compare the measured zoning of Mg in chloritoid grains of the new whiteschist samples (Fig. 11d) with a range of thermodynamically predicted zoning of Mg content in chloritoid using fractionated growth of chloritoid using the Theriak-program (de Capitani & Brown, 1987) (Fig. 13). A similar approach applied to matrix dependent garnet growth was employed by Robyr et al. (2014), as well as in more elaborated garnet growth zoning models (e.g. Gaidies et al., 2008). We interpret zoning patterns within coarse grained chloritoid (sample 19MR-03) to represent the onset of chloritoid growth during prograde metamorphism within cores having low X_Mg_, and peak metamorphism having high X_Mg_ (Figs. 11d and 13c).

Both the predicted evolving X_Mg_ content of chloritoid (Fig. 13a) and the volume% of chloritoid (Fig. 13b) are necessary to predict the zoning patterns in natural chloritoid (Fig. 13c). Unique P–T paths provide unique X_Mg_ zoning patterns during prograde metamorphism (Fig. 13a, b, d). Comparing calculated volume% zoning patterns with profiles, however, requires converting the calculated volume% to a representative radius of a 3-dimensional volume (see Appendix 1 for more details). Figure 13e shows the thermodynamically predicted X_Mg_ zoning plotted against a representative radius, which is calculated from the thermodynamically predicted volume% assuming a rectangular geometry (Appendix 1). Based on the average crystal dimensions in the natural whiteschist sample we use the measured Mg values from the second major axis of monoclinic chloritoid (represented in Fig. 13 as axis *a*).

Figures 13a, b demonstrate a range of possible prograde P–T paths incurred by the whiteschist body during Alpine metamorphism (paths (1) to (6)). We consider here the peak Alpine metamorphic conditions (1.6 ± 0.2 GPa and 585 ± 20 °C) calculated for a range of metapelitic samples analyzed in Vaughan-Hammon et al. (2021), and a P of 1.4–1.6 GPa for metagranitic assemblages (Luisier et al., 2019) to be reliable, thus we start with a prograde path from 0 °C and 0 GPa to 580 °C and 1.6 GPa (path 1). In order to reach peak metamorphic conditions for high P whiteschist lithologies calculated in this study (2.1 GPa and 580 °C), we present several possibilities for the prograde P–T evolution that represent a prograde path due to tectonic overpressure (with respect to the metagranite/metapelite path) that deviates from the standard burial path (paths 1 to 5), as well as an unrealistic high P path (path 6).

The most extreme path takes an almost isothermal prograde path from 1.6 GPa to 2.1 GPa (Fig. 13a pathway 1). The corresponding calculated zoning pattern during chloritoid growth does not agree with the measured zoning (Fig. 13e, pathway 1), due to the decrease in X_Mg_ when passing through the Ph + Ctd + Chl + Tlc + aQz stability field (Fig. 13a). This thermodynamically predicted decrease in X_Mg_ is due to the appearance of talc. We do not observe a decrease in X_Mg_ towards the rim of chloritoid grains for sample 19MR-03 related to prograde metamorphism (Fig. 13a). A retrograde decrease in X_Mg_ was observed (Figs. 10 f, g and 11d) locally due to chlorite growth within late brittle fractures in and surrounding chloritoid (red arrow Fig. 10g), which texturally overprints the peak paragenesis (Ph + Ctd + Tlc + aQz) (Fig. 10b, d). This decrease in X_Mg_, locally at the rim of chloritoid, is most likely due to a decompression P–T path from the peak assemblage without chlorite (Ph + Ctd + Tlc + aQz), to an assemblage with chlorite (Ph + Ctd + Chl + Tlc + aQz) (Fig. 13b).

The second most extreme path takes an isobaric pathway at a fixed P of 2.1 GPa (path 6). The corresponding calculated zoning pattern predicts several breaks in slope of X_Mg_ that are not comparable to the smooth increase in X_Mg_ observed in natural chloritoid zoning (Fig. 13c).

Several other potential P–T paths are shown in Fig. 13a, b that deviate from the prograde path to 1.6 GPa, which represent deviations in metamorphic conditions incurred by the whiteschist prior to reaching values that are more typical for the Monte Rosa nappe at ca. 1.6 GPa (Dal Borghi et al., 1996; Keller et al., 2004; Luisier et al., 2019; Piaz & Lombardo, 1986; Vaughan-Hammon et al., 2021). Due to the T sensitivity of Mg absorption during chloritoid growth, an isothermal P increase and deviation at lower temperatures (pathway 4 and 5), will result in minor breaks in slope of X_Mg_ values and an unrealistic X_Mg_ profile (Fig. 13e). However, these breaks in slope are small enough to be within the error range and could either be undetected or smoothed due to late stage diffusion.

P–T paths 2 and 3 do closely resemble measured X_Mg_ chloritoid zoning, which have to avoid the Ph + Ctd + Chl + Tlc + aQz stability field and the subsequent drop in X_Mg_ values (Fig. 13a). X_Mg_ zoning calculated for prograde paths 2 and 3 closely resemble (within error) measured X_Mg_ of chloritoid, especially when considering a 3-dimensional geometry (Appendix 1). Therefore, observed X_Mg_ zoning in natural samples agrees with prograde paths calculated by equilibrium pseudo-sections. It is difficult to determine accurately at which P chloritoid started to grow, because P can range significantly by ± 0.5 GPa due to the appearance of the 0.3 X_Mg_ isopleth within the chloritoid-in field (Fig. 13a).

Although many prograde P–T paths can be proposed leading to peak metamorphic conditions, our data suggests a deviation from the straight P–T path having peak P and T of 1.6 GPa and 580 °C (Fig. 13a). Such P–T path is supported by metamorphic conditions estimated for the majority of lithologies in the Monte Rose nappe, which have been calculated at ≤ 1.6 GPa.

Our results further suggest that there was no dramatic isothermal P increase for the whiteschist around peak conditions (path 1; Fig. 13), but that the whiteschist experienced a prograde P–T path with a continuous increase in both P and T (paths 2 to 5; Fig. 13). Furthermore, the measured X_Mg_ in zoned chloritoid is compatible with predictions from pseudo-section modelling, suggesting that chloritoid grew under equilibrium conditions. Consequently, our results suggest that the whiteschist exhibits a different P–T path than the metagranite and metapelite lithologies (see discussion in Sect. 5.4.).

### Alpine orogenesis: structural evolution

Numerous studies have compiled the ductile deformation history incurred by the Monte Rosa nappe, specifically within the context of the larger-scale tectonics during the Alpine orogeny (Keller & Schmid, 2001; Keller et al., 2004; Kramer, 2002; Steck et al., 2015). The earliest Alpine deformation structures reported by authors involves shearing of eclogite facies rocks and is associated with top-N nappe stacking due to underplating of Europe below Adria (e.g. Steck et al., 2015). Early ductile deformation is referred to as X_I_ by Steck et al. (2015) in the cirque du Véraz locality, and D1/D2 by Keller and Schmid (2001) and Keller et al. (2004) in the eastern, Antrona valley, region of the Monte Rosa nappe. Top-N stretching lineations, referred to as X2 in this study (Fig. 8b), correlate well with early northward directed stretching lineations reported by the aforementioned authors (Keller & Schmid, 2001; Steck et al., 2015). However, most ductile shear zones within the Monte Rosa nappe, specifically in the cirque du Véraz, do not appear to occur under high-pressure eclogite conditions, due to the lack of microstructures indicating syn-kinematic growth of high-pressure minerals. Two scenarios may explain this lack: (1) insignificant deformation during high-pressure conditions, or (2) over printing of former high-pressure microstructures under later albite-epidote amphibolite conditions. Observations of whiteschist deformation, from new samples presented in this study, do not exhibit a syn-high-pressure fabric, rather a fabric that affects the high-pressure mineralogy, which is equally not associated with greenschist mineralogy (Fig. 10a). Considering these observations, we interpret that top-N deformation structures (S2 and X2) (Figs. 6, 8a, b), represent a deformation event post-dating high-pressure metamorphism during peak Alpine conditions.

Ductile deformation associated with top-S lineations, asymmetrical folding and fold hinges plunging towards the SW (S3 and X3 of this study), correlate well with top-S D3 shearing outlined in Keller and Schmid (2001) in the eastern, Antrona valley, region of the Monte Rosa nappe, stretching lineations X_II_ and fold axis by Steck et al. (2015) in the cirque du Véraz locality, and fold axis observed by Kramer (2002) in the cirque du Véraz locality (Fig. 8b, c). S3, top-S sense of shear, and F4 asymmetrical folding styles, agree with the backfolding style of the Western Alps, which is most evident in the distorted profile of the nappe pile southwest of the Simplon line (Fig. 1, (e.g. Keller et al., 2005; Steck et al., 2015). The location of the Monte Rosa nappe in the cirque du Véraz, provides a unique opportunity to observe structurally higher regions of the larger-scale backfold geometry affecting the nappe, namely a true antiformal hinge of the Western Alps (Fig. 1c). This backfold geometry is most evident in cross section (Fig. 2), and the observations of northward and southward dipping structures associated with schistosity development (Fig. 8a). Exposure of the true hinge of late stage backfolding in the cirque du Véraz, is characterized by a more open fold geometry, compared to the tight, orogen parallel axial plane structures of the structurally lower portions of the nappe pile exposed to the NE, in the Vanzone region for example (Steck et al., 2015). By analyzing the larger scale structure of late stage backfolding (our D3), we can observe a nappe refolding geometry (e.g. Bucher et al., 2003b) characterized by asymmetrical folding whereby the northern limb has a southward directed shear sense (Fig. 7b), and the southern limb has a north directed shear sense.

### Tectono-metamorphic history of the Monte Rosa nappe

**Fig. 14:**
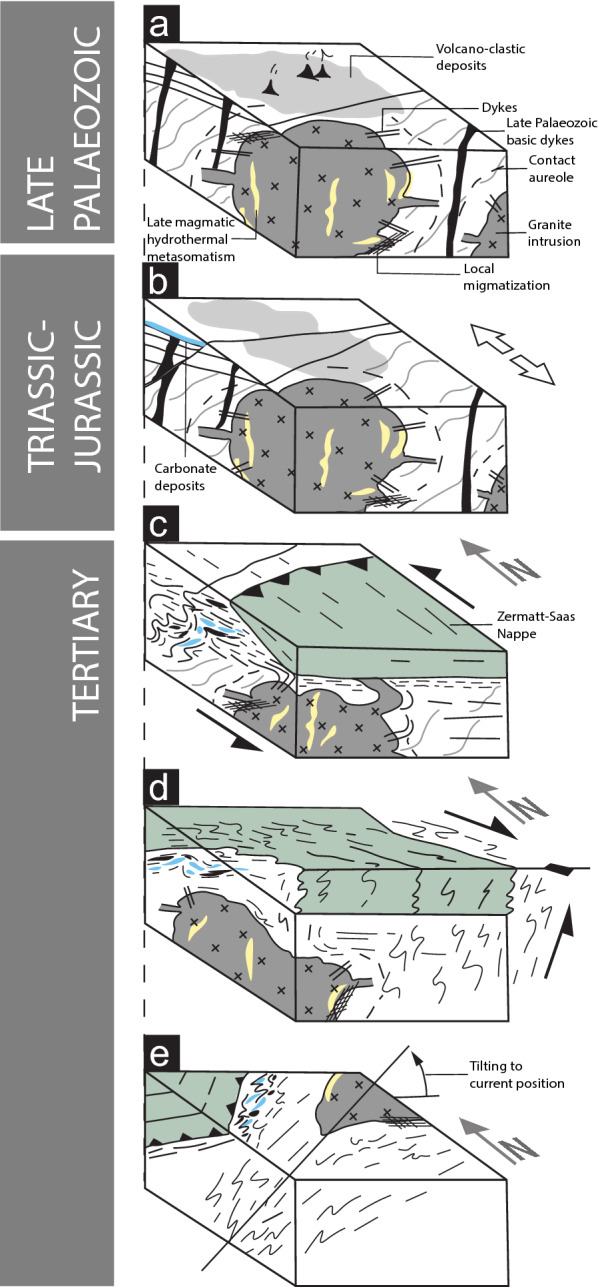
Schematic geological evolution of the Monte Rosa nappe encompassing the range of lithologies and structures exposed at the upper val d’Ayas, Italy: **a** late Paleozoic intrusion of Monte Rosa granite bodies into Variscan metapelite basement. **b** Triassic-Jurassic rifting and extension, and deposition of shallow carbonate sediments. **c** Tertiary Alpine orogenesis and over-thrusting of Zermatt-Saas ophiolite units above the Monte Rosa continental complex. **d** Late-Alpine backfolding phase. **e** Late-Alpine tilting and orogen parallel strike-slip movement, resulting in the present day position of the Monte Rosa and Zermatt-Saas nappes

Figure 14 schematically outlines the tectono-metamorphic evolution of the Monte Rosa nappe exposed in the cirque du Véraz (Fig. 2), which encompasses a range of lithologies and structures documented in this and earlier studies. This includes:

1. Variscan basement complex, including: paragneisses (Monte Rosa), Palaeozoic sediments, associated basic dykes, and volcano-clastic deposits (Furgg zone).
2. Permian-aged intrusion of granite bodies and associated dykes into Variscan basement complex. Contact metamorphism of Variscan basement close to the granite, leading to local migmatization. Fluids released by the crystallizing granite leads to late magmatic hydrothermal fluid-rock interaction producing the whiteschist protolith (Fig. 14a).
3. Triassic-Jurassic extension resulting in deposition of Mesozoic sediments (e.g. carbonates, Furgg zone) and intrusion of basic dykes (Furgg zone) (Fig. 14b).
4. Tertiary early Alpine continent collision resulting in an eclogitic metamorphic imprint at 1.6 ± 0.2 GPa and 585 ± 20 °C (affecting metapelite and metagranite lithologies), and sparse high-pressure assemblages at 2.2 ± 0.2 GPa and 560 ± 20 °C for whiteschist lithologies, potentially representing local deviations from lithostatic pressure.
5. Top-N shearing and stacking of the overriding Zermatt-Saas ophiolitic sequence (Fig. 14c).
6. Continued top-N shearing (Fig. 14c).
7. Top-S shearing and backfolding (Fig. 14d).
8. Tilting of western Alpine units to current position and orogen-parallel, brittle/ductile faulting (Fig. 14e).

### Possible explanations for P variations

This study confirms differences in estimated Alpine peak P between Monte Rosa metagranite and metapelite at 1.6 ± 0.2 GPa (Luisier et al., 2019; Vaughan-Hammon et al., 2021) and two whiteschist lenses at 2.2 ± 0.2 GPa, considering the whiteschist analyzed here and another one investigated by Luisier et al. (2019). There are several possible explanations, or hypotheses, for these P differences obtained in different lithologies, which are briefly discussed below.

Tectonic mixing (mélange), which is characteristic in numerical models of exhumation within a subduction channel (e.g. Gerya et al., 2008), is a possibility to juxtapose high P whiteschist bodies next to lower P metagranite and metapelite lithologies. However, field evidence from this study clearly shows that the association of metagranite intrusion and Variscan basement complex (mainly metapelites) of the Monte Rosa nappe exposed in the cirque du Véraz represents a structurally coherent body. Field observations also show that the whiteschists are part of the Monte Rosa nappe, since they are either fully embedded in metagranite or occur at the boundary between metagranite and Monte Rosa metapelite. Therefore, no evidence supporting tectonic mixing has been found in the field and we clearly demonstrate that the hypothesis of tectonic mélange is falsified in the studied region (Figs. 2, 3a, 4d and 6a).

It is also possible that either the peak P estimates for metagranite and metapelite or, alternatively, the estimates for the whiteschists are grossly inaccurate. If one or more of the peak P estimates were indeed considerably inaccurate, then all reported peak P estimates based on metagranitic, metapelitic and whiteschist lithologies have to be questioned, since at present it is not clear which of the peak P estimates would be grossly inaccurate. A possible explanation for which all P estimates would be accurate would be, for example, that the P estimates for the metagranite and metapelite would both indicate a retrograde P and not peak P. Since all the T estimates are similar, the retrograde P–T path from high-P whiteschist to lower-P metagranite/metapelite would be close to isothermal. Another possibility would be that both metagranite and metapelite stopped recording P at ca. 1.6 GPa due to sluggish kinetics so that both metagranite and metapelite never recorded peak P conditions. However, we have currently no indication that the P estimates for metagranite and metapelite were strongly affected by sluggish kinetics or reflect a retrograde overprint. Also, P estimates for metagranite [1.4 GPa based on Si-content in phengite and < 1.6 GPa based on absence of jadeite; Luisier et al., (2019)] and metapelite [1.6 ± 0.2 GPa based on pseudo-section modelling; Vaughan-Hammon et al., (2021)] agree within error, which rather suggests that both rocks recorded the same metamorphic peak event. Currently, we do not have any good reason to question any of the peak-P estimates.

Tectonic, or dynamic, P variations have been also proposed to explain the differences in peak P estimates (Luisier et al., 2019; Vaughan-Hammon et al., 2021). More specifically, two end-member dynamic processes have been proposed: (i) tectonically induced compressive stresses causing the shearing-off of the Monte Rosa nappe from the subducting European plate, and (ii) reaction-induced stresses due to volumetric strains during whiteschist formation (Luisier et al., 2019). Two arguments are frequently used against dynamic P variations in viscous rock for T > ca. 500 °C: (1) The rocks are mechanically too weak, that means the effective viscosity is too small, and, hence, differential stresses cannot be large so that associated dynamic P variations are negligible, and (2) Tectonic overpressure cannot occur in mechanically weak rock, only in strong rock. Concerning (1): The differential stress, and hence the dynamic P, in a viscous rock is not controlled only by its viscosity, but by the product of viscosity times strain rate. Therefore, if the strain rate is temporarily and locally significantly increased, e.g., associated with the shearing-off of crustal rocks from the subducting plate, then differential stresses can be temporarily and locally much higher than expected from average tectonic strain rates. The same applies to reaction-induced stresses and to volumetric strain rates which are related to the duration of metamorphic reactions and the associated volume changes. Concerning (2): It has been shown in several studies that mechanically weak rocks that are located between stronger rocks, either as inclusion or within a shear zone, can exhibit significant tectonic overpressure (e.g. Jamtveit et al., 2018; Moulas et al., 2014; Schmalholz & Podladchikov, 2013). This tectonic overpressure in weak rocks is simply the consequence of the force balance between strong and weak rocks. Consequently, it is mechanically possible that weak viscous rocks exhibit significant tectonic overpressure.

The newly discovered whiteschist body analyzed here complicates the attribution of P variations to simple end-member processes, since the new whiteschist body is not fully embedded in metagranite but located between metagranite and metapelite, which likely exhibit different mechanical properties (Fig. 9) (Luisier et al., 2019; Moulas et al., 2014). Although the peak P estimates for the whiteschist studied here, 2.1 ± 0.2 GPa, and for the whiteschist studied by Luisier et al., (2019), 2.2 ± 0.2 GPa, are similar, it is possible due to the error range of ± 0.2 GPa that the peak P for the two whiteschists were different by approximately 0.4 GPa. Differences in peak P between the two whiteschists would be compatible with P differences caused by differential stresses, which might have been different around the two whiteschists. If peak P was indeed different between whiteschist and metagranite/metapelite, then the minimum peak P difference, required to explain the peak P estimates within error, is 0.2 GPa (between 1.8 and 2.0 GPa). Differences in peak P ranging between 0.2 and 0.4 GPa, and related tectonic over- and under-pressure up to 0.4 GPa, are already in agreement with standard lithosphere-scale numerical models of subduction and collision (e.g. Li et al., 2010). Peak P differences > 0.4 GPa require more specific conditions, for example transient high strain rates, local mechanical heterogeneities, and/or reaction-induced stresses, which are usually not included in standard lithosphere-scale models. A more reasonable scenario that could generate dynamic P differences between metagranite/metapelite and whiteschist, would be a combination of tectonically-induced and reaction-induced differential stresses. The coupled effect of these stress states during whiteschist formation needs to be further investigated.

## Conclusions

Detailed mapping of outcrops of the Monte Rosa nappe at the cirque du Véraz reveal that the association of Monte Rosa metagranite and metapelite is a structurally coherent tectonic unit with no evidence for tectonic mixing (mélange) of these two lithologies. The studied rocks show no evidence that Alpine peak P minerals have grown during significant deformation and, hence, there is no evidence for significant deformation under peak P conditions. The structures observed during detailed mapping and the interpreted tectonic evolution of the region around the cirque du Véraz agree with the tectonic evolution reported in previous studies; both at the local scale and for the entire Monte Rosa nappe.

We describe a newly discovered whiteschist at the contact between the Monte Rosa metagranite and metapelite. Thermodynamic pseudo-section modelling indicates peak P of 2.1 ± 0.2 GPa and peak T of 560 ± 20 °C for the whiteschist. These results agree with previously reported peak P and T estimates for another whiteschist body in the same region, which was, however, entirely embedded in the metagranite. The modelling of prograde Mg-content evolution in chloritoid suggests growth of the chloritoid during a standard burial-subduction path close to equilibrium conditions. There is no indication for a dramatic, near-isothermal P increase during prograde or peak conditions.

This study confirms peak P differences between metagranite and metapelite of 1.6 ± 0.2 GPa and two whiteschists of 2.2 ± 0.2 GPa. Furthermore, this study falsifies the hypothesis that these P differences are due to tectonic mélange, that is tectonic mixing of the whiteschists from larger burial depth into less deep metagranite and metapelite. Hence, there remain currently two basic explanations for the reported P differences: (1) whiteschists, metagranite and metapelite were exposed to the same P during Alpine orogeny but one or more of the P estimates do not indicate peak conditions or are significantly inaccurate. (2) The peak P estimates are accurate within 0.2 GPa and peak P was different between whiteschists, metagranite and metapelite. We have currently no evidence that one of the peak P estimates is significantly inaccurate or that the P estimates do not indicate peak conditions. Hence, we suggest that the variation in peak P is due to differential stresses which cause local deviations from the lithostatic P. Such differential stresses can be generated by tectonic shear deformation or by reaction-induced volumetric deformation, most likely by a combination of both.

## Data Availability

Data is available on request. Fieldwork and geochemical data is available on the general-purpose open-access repository Zenodo via: https://zenodo.org/record/5519419#.YUnZwtMzaLU.

## Appendix 1: Geometrical constrains for Mg-chloritoid zoning

In order to compare the zoning of measured X_Mg_ (Fig. 13c) values in naturally occurring chloritoid with thermodynamically predicted X_Mg_ and volume proportions (Fig. 13d) of chloritoid during prograde metamorphism, assumptions need to be made on the distribution, geometry and zoning characteristics. For our natural sample 19MR-03 these assumptions include: (1) increase in volume proportion for bulk rock chemistry implies chloritoid growth, (2) synchronistic nucleation and growth of chloritoid during prograde metamorphism, (3) homogenous grainsize of chloritoids throughout specimen, (4) measured crystals have been cut through the center of the grain, (5) measured values in the core of the chloritoid grains are representative for the specimen and relate to the onset and initial growth of chloritoid, and (6) measured values at the rim of the chloritoid grains are representative for the specimen and relate to the final stages of chloritoid growth.

Unlike cubic minerals such as quartz or garnet, it is not appropriate to apply a spherical geometry when transforming thermodynamic volume proportions to measured radius for monoclinic chloritoid. 3-dimensional grainsize proportions for chloritoid in sample 19MR-03 can be simplified into 3 major axes $a$, $b$ and $c$ (Fig. 13). The measured proportions of these axes with respect to the axis of the measured profile ($a$ = 1.5 mm) correspond to $b$ = 0.8 $a$ and $c$ = 1.333 $a$. Therefore, solving for a rectangular geometry, the measured profile $a$ can be calculated via:

1 $$a= \frac{Vol\mathrm{\%}}{b c}{V}^{*}$$

where $Vol\mathrm{\%}$ is the thermodynamically predicted volume proportion of chloritoid (Fig. 13d), and ${V}^{*}$ is the normalization factor:

2 $${V}^{*}=\frac{Vm}{Vol\mathrm{\%}}$$

where $Vm$ is the measured volume using the principle axes $a$ = 1.5 mm, $b$ = 0.8 $a$ and $c$ = 1.333 $a$. Rearranging Eq. 1 in order to solve for $a$ we find:

3 $$a= \sqrt[\frac{1}{3}]{\frac{{V}^{*}Vol\mathrm{\%}}{1.0664}}$$

In order to better fit measured zoning along the chloritoid $a$ axis with thermodynamically predicted zoning in Eq. 3, we can adjust the axes proportions of chloritoid during growth. For example, only for the $c$ axis, we can include an exponential factor where $c$ = 1.333 ${a}^\frac{1}{3}$. Substituting this into Eq. 1 we derive:

4 $$a={0.82459}^\frac{1}{7}{\left({{V}^{*}}^{3}{Vol\mathrm{\%}}^{3}\right)}^\frac{1}{7}$$

The results of these variable exponential factors within the axis proportion for $c$ are presented in Fig. 13d.
